# Deciphering the viral landscape in gastric cancer: comprehensive characterization and identification of the gastric cancer virome

**DOI:** 10.1128/mbio.00551-25

**Published:** 2025-07-09

**Authors:** Dan Xiang, Shaoying Li, Jiayi Zuo, Chenchen Mao, Yangxuan Lin, Cong Long, Pingping Cai, Weiwei Liu, Xiaorong Lu, Miaofang Xiao, Wangkai Xie, Chenbin Chen, Dianfeng Mei, Kezhi Lin, Zheng Han, Xian Shen, Xiangyang Xue, Shurong Shen

**Affiliations:** 1 Wenzhou Key Laboratory of Cancer-related Pathogens and Immunity, Zhejiang International Cooperation Base for Tumor-Associated Pathogen and Host Interaction, Institute of Molecular Virology and Immunology, Department of Microbiology and Immunology, School of Basic Medical Sciences, Wenzhou Medical University 572485, Wenzhou, China; 2 Department of Medical Oncology, Wenzhou Central Hospital 223520, Wenzhou, China; 3 School of Medicine, Shanghai University, Shanghai, China; 4 Department of General Surgery, The Second Affiliated Hospital and Yuying Children’s Hospital of Wenzhou Medical University, Wenzhou, China; 5 Department of Thoracic Surgery, The First Affiliated Hospital of Wenzhou Medical University 89657, Wenzhou, China; 6 Hantai District Maternal and Child Health Hospital, Hanzhong, China; 7 Department of General Surgery, The First Affiliated Hospital of Wenzhou Medical University 89657, Wenzhou, China; 8 Department of Clinical Laboratory, Xuyong County People's Hospital, Luzhou, China; 9 Department of Medical Oncology, The First Affiliated Hospital of Wenzhou Medical University 89657, Wenzhou, China; 10 Zhejiang Key Laboratory of Intelligent Cancer Biomarker Discovery and Translation, Wenzhou Medical University 26453, Wenzhou, China; Louis Stokes Cleveland VA Medical Center, Cleveland, Ohio, USA

**Keywords:** gastric cancer, virology map, HERV-K, viral biomarkers, tumor microenvironment

## Abstract

Infectious diseases caused by pathogenic microorganisms and tumors arising from uncontrolled cell proliferation can be intricately linked through the lens of viromics. This study aimed to delineate the landscape of viral infections in gastric cancer, focusing on the relationship between pathogenic microorganisms and tumorigenesis. By conducting transcriptomic sequencing of gastric cancer tissues, we identified gastric cancer-associated viruses from raw transcriptomic data. This methodology was validated using public databases and experimental evidence, ensuring the authenticity of viral detection. Single-cell data further identified specific cells harboring these viruses and elucidated the correlation between viral gene expression and the genesis and progression of gastric cancer. Common tumor viruses, such as human endogenous retrovirus (HERV), Epstein-Barr virus (EBV), and human cytomegalovirus (HCMV), along with unique DNA viruses and bacteriophages, were detected in gastric cancer. Additionally, these viruses have been detected in both tumor and immune cells, indicating a close association between viral gene expression and the development of gastric cancer. This study advances the technique of screening viruses from transcriptomes, charting the distribution of viruses within tumors, clarifying the interplay between viruses and cancer, and identifying previously unknown viruses related to the occurrence and progression of cancer.

**IMPORTANCE:**

In our study, we have carefully examined the viral landscape in gastric cancer, supported by a series of thorough experiments that verify its reliability. Our approach has not only confirmed the presence of viruses known to be associated with cancer development but also identified a range of additional viral entities, including human cytomegalovirus (HCMV) and various herpesviruses, along with numerous bacteriophages. The high incidence of these viruses within tumor samples suggests they could be considered as potential biomarkers for early cancer detection. This method enhances our understanding of the role viruses play in cancer, which may assist scientists and medical professionals in identifying viral presence in cancers and could offer new angles for cancer prevention and the development of related measures. By identifying specific viruses linked to different cancers, we aim to improve patient outcomes.

## INTRODUCTION

Infectious diseases are caused by pathogenic microorganisms, whereas tumors are lesions resulting from uncontrolled cell growth. Approximately 13% of tumor development is associated with viral carcinogenic infections (1). For example, human papillomavirus (HPV) is a high-risk virus associated with the development of tumors such as cervical cancer (2). Moreover, approximately one-third of cancer-related deaths are attributable to lifestyle factors and chronic infections, such as those caused by *Helicobacter pylori*, hepatitis B virus (HBV), and hepatitis C virus (3). Different types of tumors can be associated with distinct viruses, with each participating in tumorigenesis and development through specific mechanisms. Oncogenic DNA viruses, such as Epstein-Barr virus (EBV), HBV, and HPV, can integrate into the host cell genome, causing genome instability and resulting in chromosomal inversions, translocations, deletions, and rearrangements, consequently generating novel fusion genes and causing dysregulated expression of host genes (4, 5). Kaposi’s sarcoma-associated herpes virus manipulates the host enzymes, CDK6 and CAD, modifying cellular nucleotide production and glucose metabolism, which facilitates viral replication as well as uncontrollable proliferation of cells, ultimately increasing the risk of tumors (6).

Gastric cancer (GC) is a common and fatal malignant tumor, with high incidence (4.9%) and mortality rates (6.8%) still prevailing in many countries (7). According to data from the World Health Organization, the incidence of GC in Asian countries is significantly higher than that in Western countries. For instance, the incidence of GC in China was ranked third in the 2022 cancer incidence statistics (7, 8), following lung and breast cancers. Each year, approximately 1.2 million new cases of GC are reported globally, with approximately 500,000 cases originating from China alone, accounting for 42% of the world’s GC incidence, which is 4–8 times higher than that in developed Western countries. *H. pylori* infection is a major risk factor for GC, particularly in Asia. Dietary habits in Asian countries may exacerbate the spread of *H. pylori*, with current infection rates reaching 50%–56%, affecting approximately 700 million people (9). In contrast, Western countries have made substantial progress in controlling *H. pylori* infection by conducting gastroscopic screenings, resulting in reduced incidence of GC. *H. pylori* is an important component of the gastric microbiome, and its abundance and diversity influence other microbial communities (10). Dysbiosis of the gastric microbiome in patients with GC has been associated with microbial communities with genotoxic potential, indicating that, in addition to *H. pylori*, other microbial communities may also contribute to the persistent inflammation of the gastric mucosa and the development of GC (9). The occurrence and progression of GC are closely related to the dysregulation of the gastric microbiome. The microbiome primarily protects the gastrointestinal tract from exogenous pathogens and potentially harmful endogenous microorganisms by directly competing for limited nutrients and modulating host immune responses. Dysregulation of normal microbial communities increases the risk of pathogenic infection, overgrowth of harmful pathogens, and inflammatory responses (11). The association between human cytomegalovirus (HCMV), EBV, and GC adds complexity to the etiology of this disease, underscoring the importance of in-depth studies on cancer-associated viral distribution and classification (12).

The rapid development of next-generation sequencing (NGS) technology has created unprecedented opportunities and challenges in viral analysis. Due to its high throughput, low cost, and wide range of applications, NGS has become an essential tool for studying viral diversity, evolution, and host interactions. However, factors such as biases in library construction, algorithm selection, and threshold settings during data analysis can lead to false-positive results, potentially causing misinterpretations of viral infection status. Therefore, a thorough examination of the strengths and limitations of NGS in viral analysis is crucial. This will not only help optimize sequencing strategies but also establish a more reliable foundation for subsequent viral monitoring and control.

Despite the growing number of studies on tumor virology, most focus on highly expressed or well-characterized viruses, neglecting comprehensive screening and experimental validation of the entire spectrum of viral candidates. In this study, we employed a computational workflow to extract viral data from RNA-Seq data sets of GC, identify and eliminate experimental contamination, and experimentally verify and quantify the association among GC, *H. pylori,* and microbiome composition. This approach revealed the relationship between viral abundance and the clinical incidence of GC, thus mapping the viral landscape within the disease context.

## RESULTS

### Depiction of virome landscape in GC-based bulk RNA-seq

**Fig 1:**
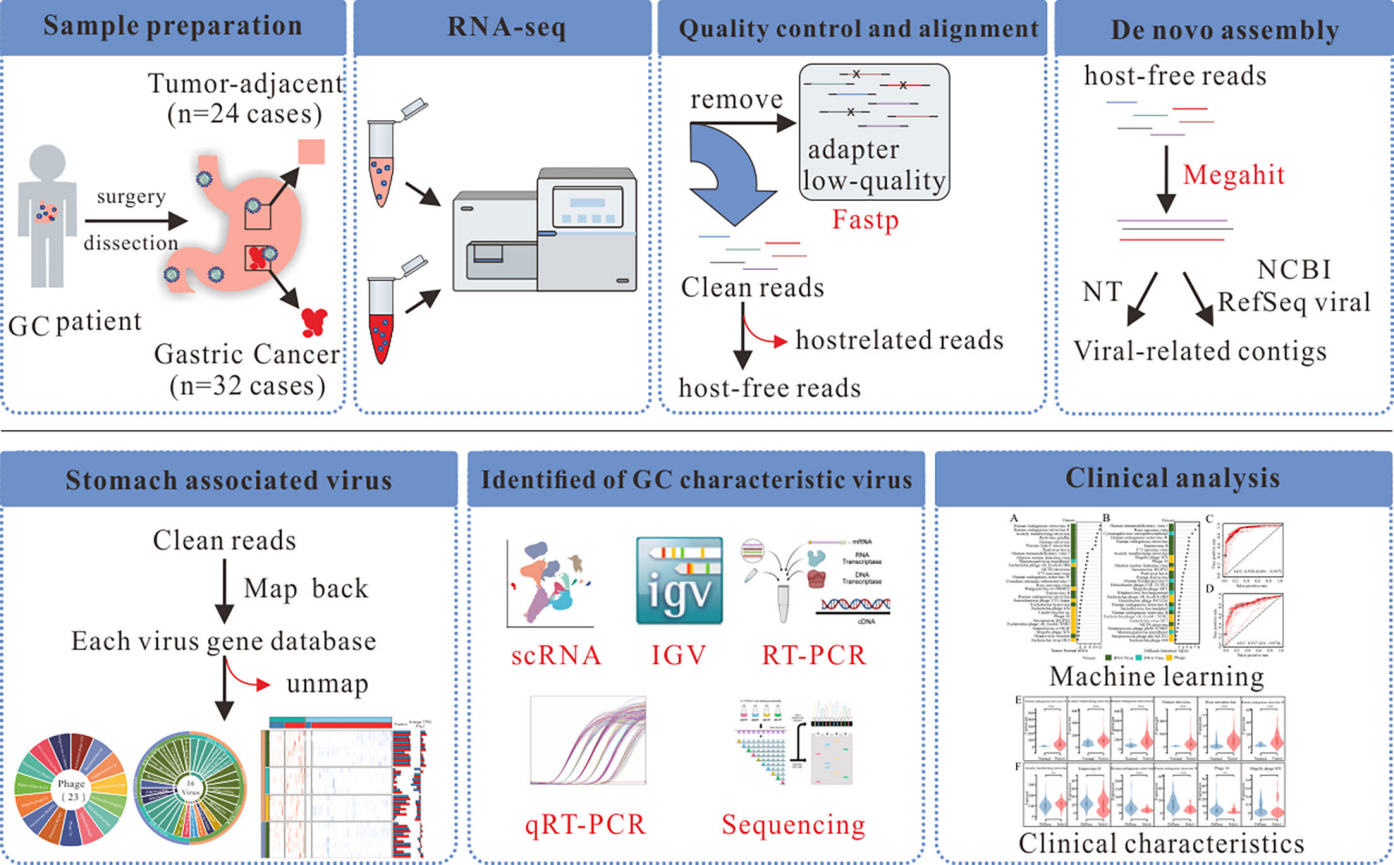
Flowchart summarizing the main design of the present study.

**Fig 2:**
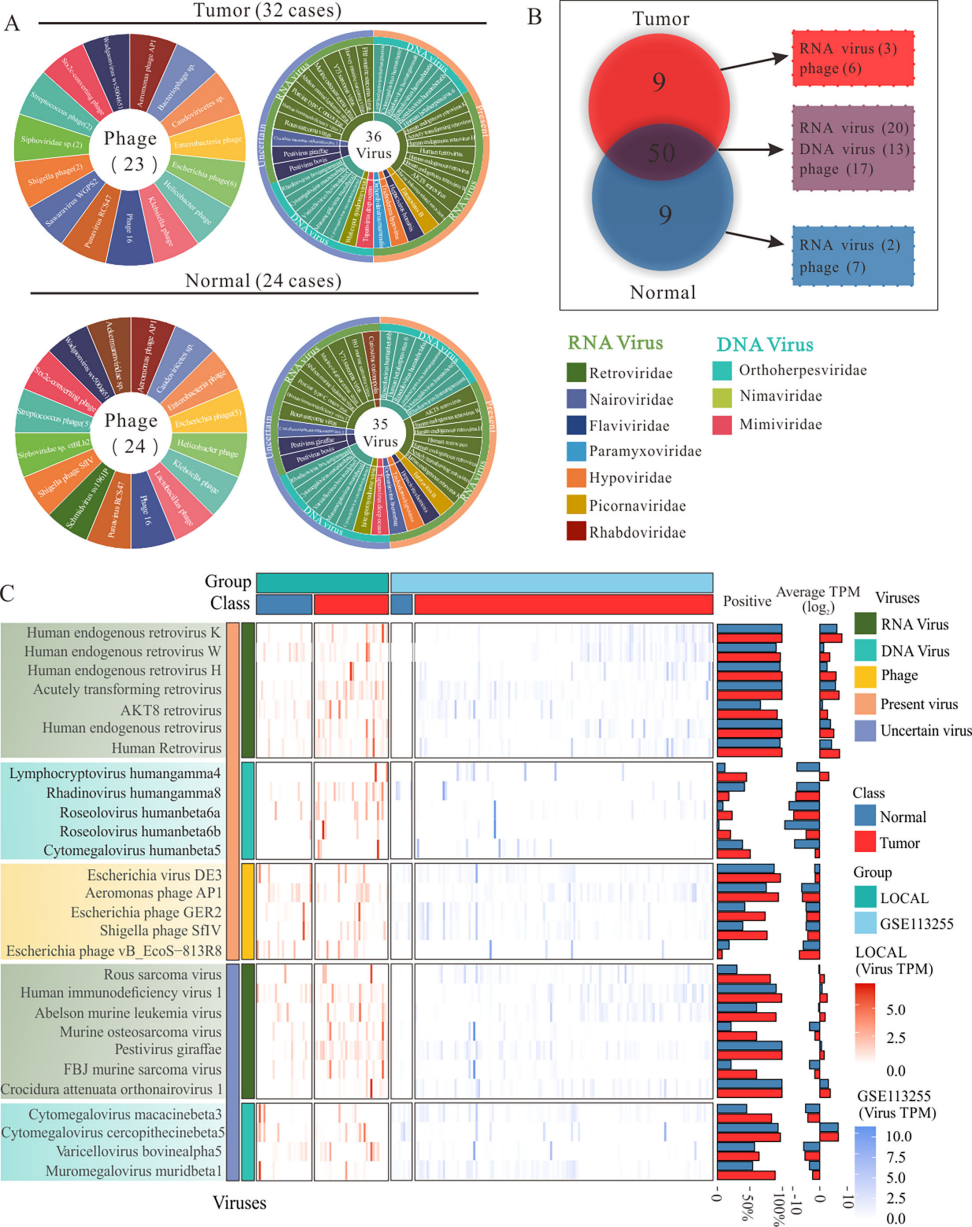
Depiction of the virome landscape in GC. (**A**) Overview of virus classification in LOCAL, from family to genus. Different families are labeled in different colors. Tumor phage (*n* = 23), tumor RNA and DNA virus (*n* = 36), normal phage (*n* = 24), normal RNA and DNA virus (*n* = 35). (**B**) Nine were exclusively present in the GC tissues and adjacent tissues, and 47 types of viruses were detected in both the GC tissues and adjacent tissues. (**C**) Heatmap based on TPM of 28 viruses with high expression levels of two transcriptome data sets, with 17 viruses classified as Present and 11 viruses classified as Uncertain, and the total positive rate and total reads of the virus in all data set samples. The LOCAL includes gastric cancer (*n* = 32 cases) and tumor-adjacent (*n* = 24 cases), GSE113255 includes gastric cancer (*n* = 128 cases) and tumor-adjacent (*n* = 9 cases).

The graphical flow chart outlines the main design of this study (Fig. 1). To map the landscape of GC viromes, LOCAL and LOCAL-12G RNA-seq cohorts (32 GC samples and 24 tumor-adjacent samples) were used. Not only errant alignment viruses but also quality control indicators such as phiX and Dickeya phage phiDP23.1 were removed. Additionally, some viruses that are known not to infect humans were also removed. After summarizing the types and read counts of the viruses, we identified 68 viral types, including 13 DNA viruses, 25 RNA viruses, and 30 phages. The presence of 7 DNA viruses and 12 RNA viruses in stomach tissue remains uncertain. The sequences obtained are accurate and not attributable to vector contamination. However, given the nature of these viruses, it is plausible that they could potentially infect humans (Fig. 2A). Among these, nine viruses were exclusively present in the GC tissues, while another nine were found only in the adjacent normal tissues. Specifically, Siphoviridae sp. ctHip2, Bacteriophage sp., Helicobacter phage Pt5322G, Sawaravirus WGPS2, Escherichia phage Cyrano, Shigella phage SfX, Orthorubulavirus mammalis, Murine leukemia-related retroviruses, and Harvey murine sarcoma virus were detected only in the gastric cancer tissues. In contrast, Streptococcus phage phi-SsuSSJ28_rum, Lactobacillus phage phiadh, Streptococcus phage phiSC070807, Helicobacter phage COL 23-PUJ, Schmidvirus sv1961P, Ackermannviridae sp., Streptococcus phage IPP61, Orthonairovirus haemorrhagiae, and Curiovirus curionopolis were identified only in the adjacent normal tissues. Additionally, 50 types of viruses were detected in both the GC tissues and adjacent tissues (Fig. 2B). In regular transcriptome sequencing, reads that align to phages might be due to the phages’ active transcription within the sample or their integration into the host genome, prompting viral gene expression. However, such alignment isn’t conclusive for determining phage abundance. Compared with public data sets (GSE41476 and GSE113255), DNA viruses (Lymphocryptovirus humangamma4 [EBV] and Cytomegalovirus humanbeta5 [HCMV]) and RNA viruses (human endogenous retrovirus [HERV], acutely transforming retrovirus [LNras*SN acutely transforming retrovirus]) were identified as GC-characteristic viruses, with a higher positive rate and abundance in the GC (Fig. 2C, Fig. S1).

### Virus expression in GC cell subtype

**Fig 3:**
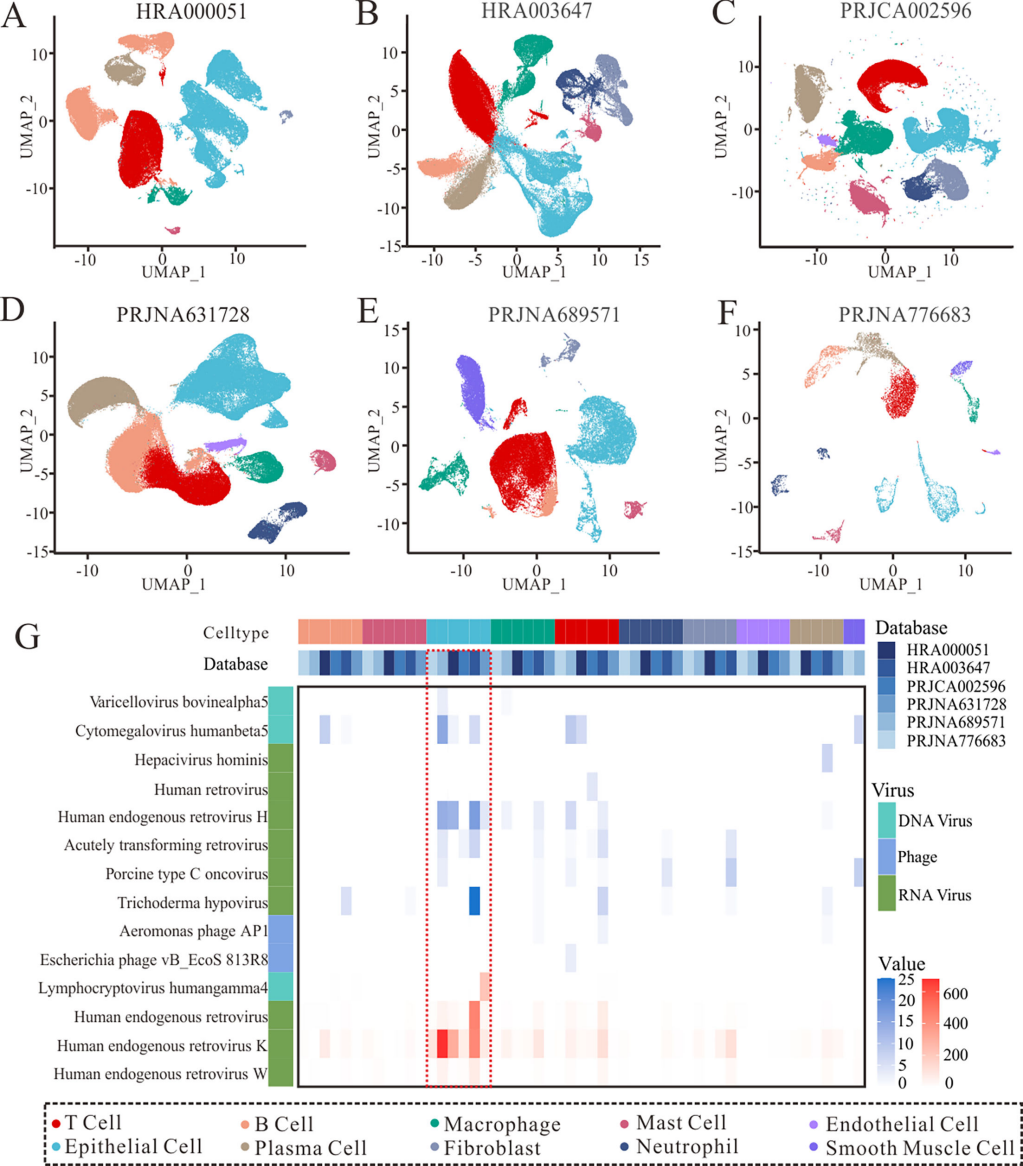
Single-cell transcriptome shows the cell type and number of cells in which the virus is present before quality control. (**A-F**) UMAP dimensionality reduction and manual annotation of six single-cell transcriptome data sets before quality control. (G) Heatmaps based on cell type and number of cells in which the virus is present.

To further determine whether the viral landscape differed between the different cell types of GC, we integrated six publicly available single-cell RNA sequencing (scRNA-seq) data sets. Clustering analysis and manual annotation using known marker genes (Table S3) were performed, and monocytes, neutrophils, B cells, T cells, natural killer (NK) cells, and epithelial cells were identified from each data set (Fig. 3A through F). Similar to the results of Bulk RNA-seq, our analysis showed that the detection of HERV, HERV-K, HERV-W, and EBV was particularly prominent across the different cell subtypes of GC, with the most significant concentrations in epithelial cells, followed by macrophages, and T and B cells (Fig. 3G, Fig. S5). In contrast, the quality control results with and without implementation show minor variations (Fig. S6). Our exhaustive examination revealed 14 unique viruses with different cellular contexts.

An in-depth analysis of recent viral discoveries revealed the ubiquitous presence of viruses, extending from immune to non-immune cells. Retroviruses have been frequently detected across 10 different cell types within six scRNA-seq data sets, encompassing B cells, dendritic cells, endothelial cells, epithelial cells, macrophages, neutrophils, T cells, NK cells, smooth muscle cells, and tissue stem cells, exhibiting high expression levels. HCMV was identified in three single-cell data sets, and its presence was noted in epithelial cells, B cells, T cells, and neutrophils. EBV was found in five distinct cell types: epithelial cells, tissue stem cells, NK cells, T cells, and B cells, across three datasets. These results underscore the dependability of single-cell analyses. Furthermore, our single-cell analysis not only corroborated the association between known viruses and GC but also identified the potential involvement of other previously overlooked viruses, such as acutely transforming retroviruses, in the development of the disease (Fig. 3G). However, after applying quality control protocols, the detection of HCMV and EBV in epithelial cells and tissue stem cells was markedly diminished compared to that in unprocessed samples (Fig. S6). Notably, while Escherichia phage and Aeromonas phage were detectable in T cells, the low abundance of other bacteriophages rendered them undetectable by single-cell sequencing, highlighting the limitations imposed by the viral load in such studies.

### Gene expression characteristics and identification of GC-characteristic virus

**Fig 4:**
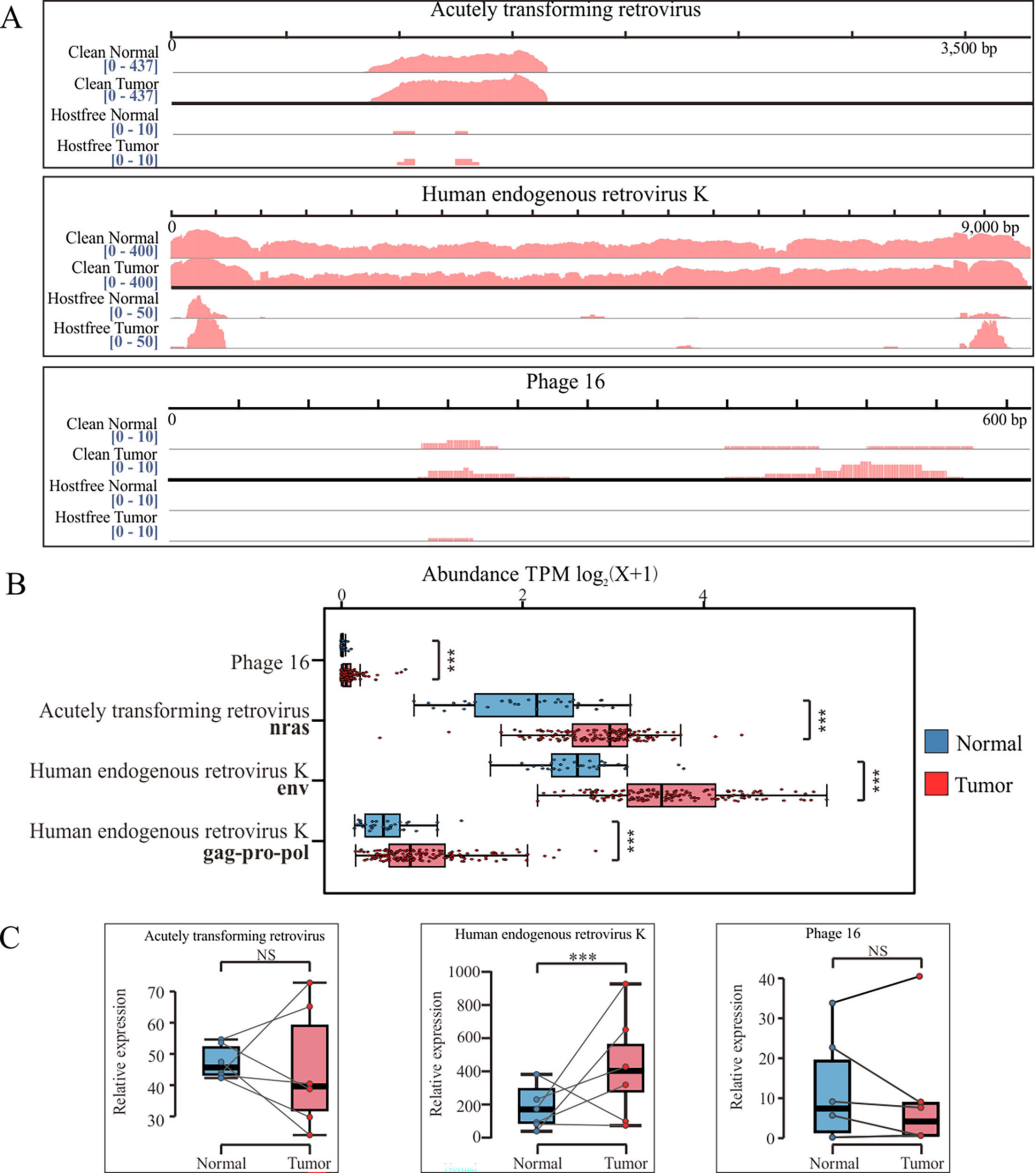
Detection of three viruses in gastric cancer tissue and adjacent tissues. (**A**) IGV software visualizes the mapping results of host-free reads and clean reads for three viruses, all of which can be aligned to multiple locations. (**B**) Differential expression of four genes from three viruses in gastric cancer tissue and adjacent tissues. Obtaining precise reads based on a methodology after building a virus gene library. (**C**) The mRNA levels of four viruses in GC tissues and the corresponding adjacent normal gastric tissues were determined by qRT-PCR. *P < 0.05, **P < 0.01, ***P < 0.001.

The abundance, positivity rate, total reads, and coverage of the 28 highly expressed viruses were analyzed, with 17 viruses classified as Present and 11 viruses classified as Uncertain (Fig. 2C and 4; Fig. S2). As demonstrated by the Integrative Genomics Viewer (IGV), most viral genes can be accurately mapped. The abundances of bacteriophages were relatively low and varied across samples. Most of the retroviruses and DNA viruses were more frequently detected in gastric cancer samples compared with tumor-adjacent samples. Within the category of DNA viruses, the detection rate of most herpes viruses, including HCMV and EBV, was relatively low, and the levels of viral abundance varied considerably. In some samples, the abundance of HCMV and EBV notably exceeded that of retroviruses, enabling mapping of the entire HCMV genome. This observation highlights the potential influence of HCMV on the GC viral landscape.

The 10 viruses with the highest genome coverage were identified. Three of these were confirmed by reverse transcription-PCR (RT-PCR) ( Fig. 3A and 4): acutely transforming retrovirus, HERV-K, and Phage 16. Feline leukemia virus primarily infects felines and isn’t transmissible to humans. IGV alignment showed only a short sequence (100 bp) with high homology to the human genome, so we regard it as a contaminant. Porcine type-C oncovirus, with pigs as its main host, may potentially infect humans. Our detection revealed its full-length sequence, only part of which is homologous to the human genome. Thus, we posit it might be present in GC (Fig. S3B). However, the PCR results for three other viruses, Pestivirus tauri, hepatitis E virus, and Phocid orthoreovirus 1, did not show any bands (Fig. S3C). Notably, both the tissues and blank controls of Punavirus P1 and Dickeya phage phiDP10.3 displayed segments that aligned with theoretical expectations (Fig. S3D). Using genes of these viruses, the expressions of Phage 16, acutely transforming retrovirus, and HERV-K were observed to be higher in tumor tissues than in normal tissues (Fig. 4B). Furthermore, quantitative reverse transcription-PCR (qRT-PCR) also confirmed that HERV-K was more highly expressed in GC (Fig. 4C). However, due to their low expression levels, Phage 16 and acutely transforming retrovirus did not show significant differences. Generation sequencing confirmed that the designed primers for these three viruses were specific.

### Relationship between viral composition and clinical characteristics of GC

**Fig 5:**
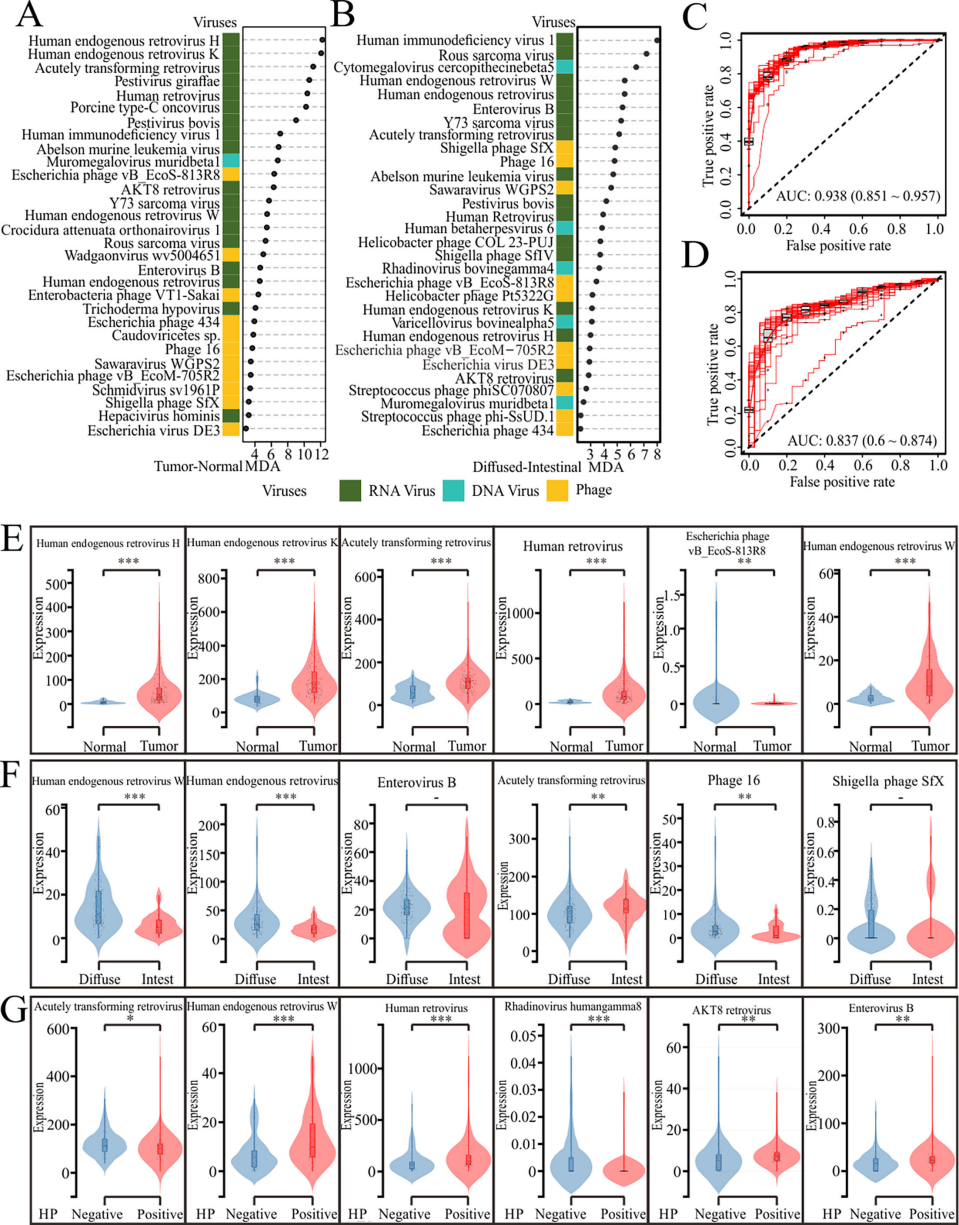
Performance of virome markers in the diagnosis of GC patients. Dot plot showing the average importance scores from 1,000 iterations of the discovery model fit and ranking of the most discriminatory virome species-level markers identified by Random Forests-based backward feature selection in the GC patients and adjacent tissues cohort (**A**) and Lauren cohort (**B**), focusing on the classification of GC subtypes. Red line represents the average true and false positive rates in the GC patients and adjacent tissues cohort (**C**) and Lauren cohort (**D**), with individual rounds of 10-fold model cross-validation depicted by out dots and summarized by boxplots. (**E**) Clinical characteristics of patients with GC show significant differences in the top six viral species within the Present virus category when comparing tumor tissues to adjacent normal tissues. (**F**) Analysis of the top six viral species in the Present virus category for their potential to distinguish between diffuse-type and intestinal-type GC. (**G**) Significant differences in the six viral species in the Present virus category between *H. pylori*-positive and *H. pylori*-negative samples. *P < 0.05, **P < 0.01, ***P < 0.001.

To determine the potential of viral taxa in distinguishing disease states, we used a backward feature selection approach based on the random forest algorithm to identify a minimal set of 30 viruses from both the LOCAL-6G and public database data sets. This method maximized the separation between GC patients and adjacent tissues (Fig. 5A) and between different Lauren classifications (Fig. 5B). In the GC patients and adjacent tissues cohort, HERV-H and HERV-K exhibited the highest mean fold increase, indicating their potential significance in the disease context (Fig. 5A). The analysis resulted in an estimated out-of-bag (OOB) error rate of 25.9%, which is a measure of the model’s performance during the training phase. The predictive model achieved an accuracy of 0.938 with a confidence interval (CI) of 0.851–0.957, indicating a high level of classification accuracy (Fig. 5C). Additionally, the training and test sets showed an accuracy of 0.88, further validating the robustness of the model. When combined with the clinical characteristics of GC, the top six viral species in the Present virus category, such as HERV-H (*P* < 0.001), HERV-K (*P* < 0.001), acutely transforming retrovirus (*P* < 0.001), human retrovirus (*P* < 0.001), Escherichia phage (*P* < 0.01), and HERV-W (*P* < 0.001) were significantly different in GC patients and adjacent tissues (Fig. 5E).

In the Lauren cohort, HERV-W exhibited the highest mean fold increase, indicating its potential significance in the disease context. The analysis resulted in an estimated OOB error rate of 28.9%, which is a measure of the model’s performance during the training phase. The predictive model achieved an accuracy of 0.837 with a CI of 0.6–0.874, indicating a high level of classification accuracy (Fig. 5D). Additionally, the training and test sets showed an accuracy of 0.86, further validating the robustness of the model. When combined with the clinical characteristics of GC, the top six viral species in the Present virus category were analyzed for their potential to distinguish between diffuse-type and intestinal-type GC. The results showed that HERV-W (*P* < 0.001), human endogenous retrovirus (*P* < 0.001), acutely transforming retrovirus (*P* < 0.01), and Phage 16 (*P* < 0.01) were significantly higher in diffuse-type GC compared to intestinal-type GC. However, Enterovirus B (*P* > 0.05) and Shigella phage SfX (*P* > 0.05) did not show significant differences between the two types.

Upon comparing the viral abundance in *H. pylori*-positive and -negative samples, significant differences were observed for several viruses, including acutely transforming retrovirus (*P* < 0.05), HERV-W (*P* < 0.001), human retrovirus (*P* < 0.001), Rhadinovirus humangamma8 (*P* < 0.001), AKT8 retrovirus (*P* < 0.005), and Enterovirus B (*P* < 0.005). These findings suggest that the presence of *Helicobacter pylori* may influence the viral composition within the gastric environment. Further investigation is needed to elucidate the mechanisms underlying these differences and their potential implications for gastric health and disease.

### HERV-K is embedded in human genes and plays a crucial role

**Fig 6:**
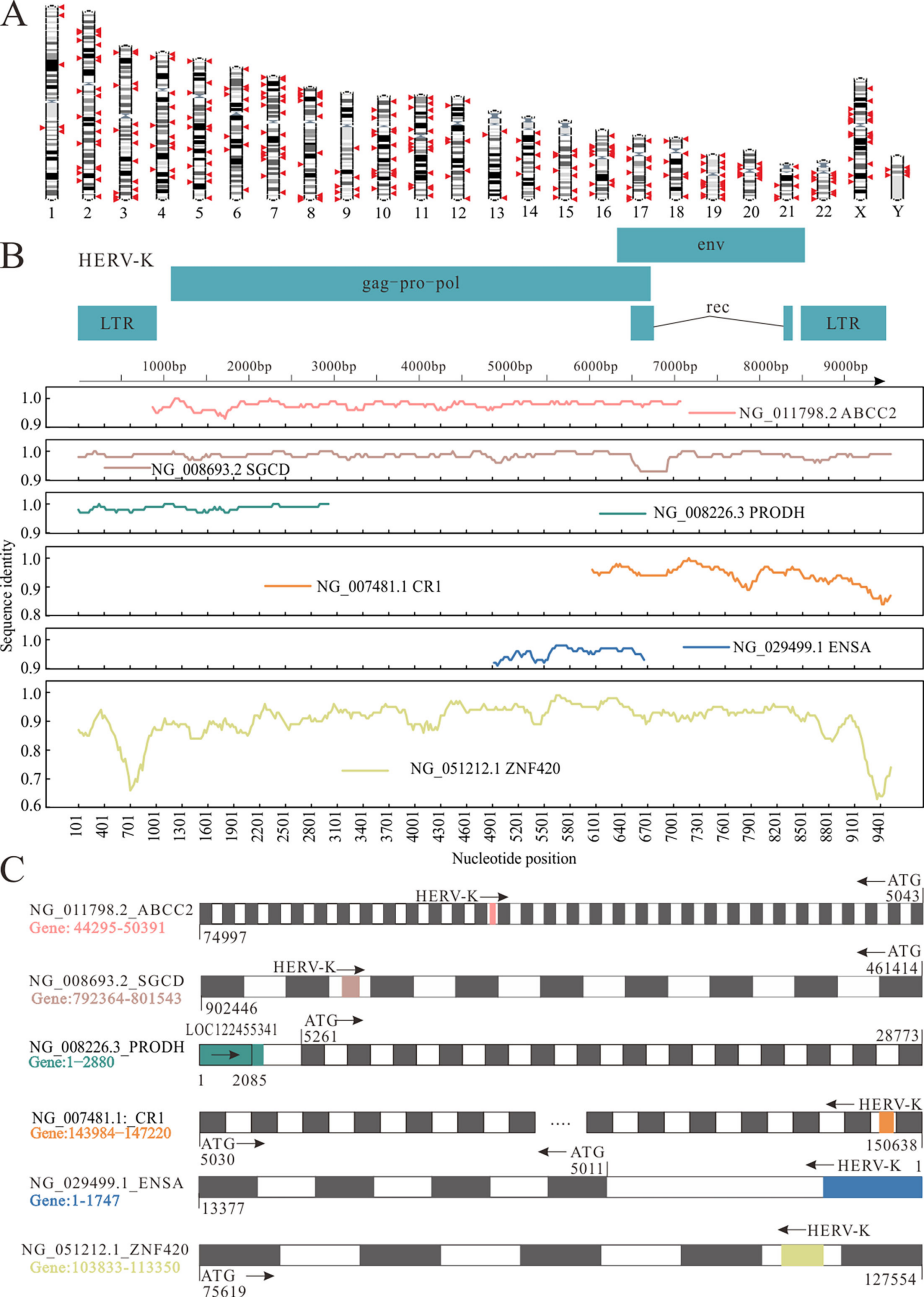
Homology between HERVs and human. (**A**) All HERV elements (red arrows) are displayed on the human karyotype (https://asia.ensembl.org/index.html). (**B**) Which base segment of a human gene is homologous to HERV-K ATG: starting codon. (**C**) The top is the composition diagram of HERV-K, and the bottom is the homologous region between HERV-K and human genes. Nucleotide position: HERV-K nucleotide site, sequence identity: homology rate.

Upon using IGV to visualize virus-mapped reads, we observed significant differences in read coverage and transcript abundance of clean and host-free reads of HERVs. The result of read coverage in clean reads spanned the whole genome, whereas host-free data spanned only a few base segments. Therefore, HERV is embedded in the human genome and regulates it to perform certain functions. Homologous sequences of HERV in humans were identified by clean reads mapping, but not by host-free unmapped reads. Figure 6A illustrates the locations of all HERV sequences in the human genome that are homologous to those found in public databases. It can be observed that homologous segments are present on each chromosome.

Given the importance of HERV-K in this study, we conducted a detailed analysis of the gene locus. This in-depth analysis allowed us to elucidate the significant differences between various HERV-K loci. All human gene fragments with notable sequence homology with HERV-K were identified (Table S4). Notably, four of these homologous regions were nested within introns (reverse: *ABCC2*, *SGCD*, *CR1*, and *ZNF420*), whereas the other two were located in promoter sequences (forward: *PRODH* and *ENSA*) (Fig. 6B). Initial comparative analysis using NCBI BLAST highlighted these genetic parallels, establishing a stage for more comprehensive investigation into their functional significance and evolutionary origins. *SGCD* and *ZNF420* contain the entire HERV-K viral genome, whereas *ABCC2*, *PRODH*, *CR1*, and *ENSA* contain only a fragment. Except for *ZNF420*, the homology rates of the other five human genes with HERV-K were high (Fig. 6C). The relationship between HERV-K and the five homologous genes requires further verification. The function of HERV-K homologous fragments can be very complex, and the current understanding of the role of these fragments in the genome remains limited. Future studies are needed to further explore the specific mechanisms of action and biological significance of these fragments.

## DISCUSSION

The evolution of virological research has introduced new perspectives for understanding the etiology of GC. Sequencing technologies offer powerful tools for dissecting the complex interplay between viral and host gene expression in GC. Given that the standardized annual incidence rate of all cancer types is increasing by an average of 1.4% (8), the role of viruses as etiological agents of cancer cannot be overlooked. However, the limitations inherent in transcriptome sequencing and alignment techniques can lead to false positives. Concurrently, certain GC-associated viruses, characterized by low abundance or unique sequence features, may evade detection. Therefore, novel methodologies must be designed for comprehensively screening and validating viruses associated with GC, as well as investigating their correlation with the disease’s occurrence and progression (13–15).

Our research identified numerous DNA viruses, RNA viruses, and bacteriophages in GC. Herpesviruses and retroviruses were the most prevalent DNA and RNA viruses, respectively (16, 17). These viruses were further confirmed through experimental validation. RNA viruses such as HERV, Flaviviridae, Hepeviridae, Nairoviridae, and Picornaviridae are present in mammals (18). However, in our study, Flaviviridae and Hepeviridae were not successfully verified. DNA viruses, including members of the Mimiviridae and Phycodnaviridae families, have been identified in the intestinal mucosa (19). Additionally, studies have confirmed the presence of HCMV (20) and EBV (4, 16) in GC tissues, while HPV and HBV have not been detected in GC patients. The Spinareoviridae virus could also not be experimentally verified. Phages, such as the Streptococcus phages, Enterobacteria phages, and members of the Siphoviridae family, were also found in the gut microbiota.

According to these studies, HERV is a retroviral sequence in the vertebrate genome that originated from the integration of ancient invasive exogenous retroviruses. When retroviruses infect host cells, they are first reverse-transcribed into double-stranded DNA and then integrated into the host genome. Exogenous retroviral DNA integrated into germline cells may become an ERV. During evolution, most integrated retroviruses have lost their ability to express viral genes (21). HERVs account for 8% of the human genome, with more than 98,000 HERV elements identified on human chromosomes (22). Single-cell sequencing has revealed the presence of retroviral genes in all cells. HERVs can be classified into three categories and 11 supergroups. Among these, the acutely transforming retrovirus that carries the oncogene v-jun and the Akt gene in the P13k-Akt signaling pathway is homologous to the AKT8 proto-oncogene (v-Akt).

HERV-K had high average importance scores for GC diagnosis and was significantly higher in diffuse GC. This suggests that viral infection may be a significant trigger for GC development and may further aggravate its progression. Conversely, the progression of GC may promote viral infections. HERV-K retains a relatively complete open reading frame and has potential biological activity (23). Some genes derived from HERVs are involved in normal human physiological processes, such as placental formation and embryogenesis (24). HERVs also play a role in regulating the human immune system and in the occurrence of various diseases, including type 1 diabetes (25), nervous system diseases (26), and other autoimmune diseases (27). Abnormal HERV expression has been detected in multiple cancers, including melanoma (28) and ovarian cancer (29). We selected HERV-K for homology analysis and identified six human genes homologous to HERV-K, among which *PRODH*, *ABCC2*, and *SGCD* have been previously reported. *PRODH* functions as an enhancer under low methylation conditions and binds to the transcription factor SOX2 to upregulate *PRODH* expression in the hippocampus (30). We propose that homologous sequences of *ENSA* also regulate *ENSA* expression. Although the function of the homologous sequences located within the introns of the other four genes is unclear, we speculate that they may encode lncRNAs that exert functional effects. These data suggest that HERV-K is a risk factor for autoimmune diseases and cancer. Notably, while current technology can detect the HERV-K virus in the human body, its complex role in the host genome and its potential impact on human diseases remain elusive.

DNA viruses also play a significant role in the pathogenesis of GC, with Herpesvirales being predominant. The high positivity rate and variable expression levels of various herpesviruses across different GC samples highlight their potential impact on cancer development. Notably, HCMV and EBV have been identified in GC tissues, suggesting their significant association with the disease. Approximately 10% of GC cases are associated with EBV. EBV-positive GCs (EBVaGCs) exhibit a unique molecular signature, including high levels of LMP1 and LMP2 expression, which are absent in EBV-negative cancers. These distinctive attributes indicate a potentially crucial role for EBV in EBVaGC tumorigenesis (4). The presence of EBV in both B cells and epithelial cells in GC suggests that EBV may influence cancer progression through multiple cellular pathways. It was initially believed that HCMV was only found in fibroblasts and immune cells of GC tissues. However, recent studies have detected HCMV in the epithelial cells of GC, indicating a broader role for HCMV in the disease than previously understood. The discovery of HCMV in these cells, along with the consistent presence of EBV, aligns with previous research outcomes and validates the credibility of single-cell analysis. Furthermore, quality control studies have confirmed the presence of these viruses in gastric epithelial cells. This indicates their potential role in accelerating GC progression, suggesting that they may not only be a consequence of the disease but could also contribute to its development and progression. Understanding the interactions between DNA viruses, particularly Orthoherpesviridae, and GC is essential for developing targeted therapies and diagnostic tools. Further research is needed to fully elucidate the mechanisms by which these viruses contribute to GC and to assess their potential as therapeutic targets.

Various bacteria and bacteriophages have also been identified in GC tissues, contributing to the complexity of the tumor microenvironment. While *H. pylori* is a well-known bacterium associated with GC, other bacteria such as *Enterobacteria, Streptococcus,* and *Pseudomonas* have also been identified (9). When comparing the abundance of viruses in samples that are *H. pylori* positive and negative, it was found that the abundance of certain viruses was significantly higher in *H. pylori*-positive samples. These findings suggest that the presence of *H. pylori* may influence the viral composition within the gastric environment (Fig. 5G). Further investigation is needed to elucidate the mechanisms underlying these differences and their potential implications for gastric health and disease. The genera *Escherichia*, *Peptococcus*, *Klebsiella*, and *Bacteroides* are known to enter a dormant state in the stomach due to its acidic environment (9). This discovery suggests a broader microbial influence on the disease than previously recognized. Under certain conditions, these bacteria may become metabolically active, potentially influencing the tumor microenvironment. Additionally, 30 of the 68 virus species identified were bacteriophages, including the Phage 16, Enterobacteria phage, Siphoviridae phage, Streptococcus phage, and Pseudomonas phage. According to the information in the NCBI database, the sequence of Phage 16 shows that its host is human, and its genome contains large segments of sequences that are homologous to the host. This phenomenon suggests that Phage 16 may not be a typical phage associated with gastric cancer, but rather may have undergone some form of integration or recombination with the host genome. This homology may imply interactions between Phage 16 and the host genome during evolution, or its sequence may have been mistakenly identified as phage-derived in the sample. The systemic presence of these phages could potentially affect human health by interacting with the immune system and contributing to the development and progression of GC. Understanding the roles of bacteriophages and the bacterial landscape in GC is supported by emerging research, highlighting the need for further investigations into their interactions and influence on cancer biology and treatment (31).

We observed significant differences in read coverage and transcript abundance between clean and host-free viral reads. Although RNA viruses have a high positivity rate and are abundant in GC, most are unverifiable or undetectable, and retroviruses are often integrated into the human genome. While DNA viruses are detected at a lower prevalence and with fewer reads, IGV analysis demonstrates consistent read coverage and transcript abundance for both clean reads and host-free reads of these viruses. This indicates that even when present in trace amounts, DNA viruses and phages can be accurately identified, and their transcription levels can be assessed within the sampled tissue. However, due to its low expression level, the phage cannot be experimentally validated. Further detection of its bacterial host is required to clarify this. A thorough delineation of the GC virome involves not only the exploration of GC-associated viruses—HERV (16, 23, 32), EBV, and HCMV—viruses related to *H. pylori* infection, as well as the presence and activity of other previously overlooked viruses in the development of GC. It is undeniable that our study has certain limitations in viral detection, such as the inability to encompass viruses with extremely low abundance, those in a dormant state, or those lacking polyA tails. In the future, we plan to utilize whole-genome and transcriptome sequencing technologies to conduct a more in-depth analysis of the relationship between viruses and the onset and progression of tumors.

In summary, our approach for identifying viruses within human transcriptomes has been rigorously validated through a comprehensive suite of experiments, proving it to be a reliable technique. Our methodology will aid researchers and clinicians in identifying the presence of viruses across various cancer types, discerning those implicated in the onset and progression of cancer, and offering novel perspectives for cancer prevention strategies. This systematic method of viral detection not only enhances our understanding of the viral influence on carcinogenesis but also contributes to the advancement of preventive measures and therapeutic approaches. By identifying specific viruses associated with different cancers, we can better tailor strategies to mitigate risks and improve patient outcomes globally.

## MATERIALS AND METHODS

### Data sets and tissue sample collection

Gastric cancer (*n* = 32 cases) and tumor-adjacent (*n* = 24 cases) tissues were collected from The First Affiliated Hospital of Wenzhou Medical University (Wenzhou, People’s Republic of China). Samples were stored at −80°C until transcriptome sequencing. Meanwhile, 10 paired gastric cancer and tumor-adjacent samples were collected from The Second Affiliated Hospital of Wenzhou Medical University (Wenzhou, People’s Republic of China). Once collected, specimens were deposited into tubes and preserved in liquid nitrogen immediately until further use. The histopathological diagnosis of gastric tumor and paired non-tumor specimens was confirmed following surgery by the pathological department of The First Affiliated Hospital of Wenzhou Medical University and The Second Affiliated Hospital of Wenzhou Medical University. The present study protocol was approved by the Human Research Ethics Committee of The First Affiliated Hospital of Wenzhou Medical University and The Second Affiliated Hospital of Wenzhou Medical University, and written informed consent was provided by all participants.

Publicly available transcriptomic profiles with accession numbers GSE41476 (gastric cancer = 3 cases and tumor-adjacent = 2 cases) and GSE113255 (gastric cancer = 128 cases and tumor-adjacent = 9 cases) were downloaded from the NCBI Gene Expression Omnibus (GEO) database. One hundred thirty-seven gastric cancer samples with complete clinical information in GSE113255 were used for further analysis.

### RNA isolation and transcriptome sequencing

Total RNA was extracted using TRIzol Reagent (Thermo Fisher Scientific, Carlsbad, CA, USA). rRNA was removed from the total RNA using a ribosomal RNA removal kit. mRNA was then purified from the remaining RNA using poly-T oligo-attached magnetic beads. The purified mRNA was fragmented and converted into cDNA. Samples collected from the First Affiliated Hospital of Wenzhou Medical University were used for transcriptome sequencing. RNA was quantified and then used for library preparation. Sequencing libraries were generated using the NEBNext Ultra Directional RNA Library Prep Kit for Illumina (NEB, USA) following the manufacturer’s recommendations. In order to reduce index hopping, the strategy “prepare dual-indexed libraries with unique indexes” was used, and unique dual indexes (UDI) were attached to the ends of the libraries for cross-verifying the indexes. Libraries were sequenced on the Illumina Novaseq 6000 platform (PE150, Illumina), LOCAL group with ~6 Gb (all the samples) and LOCAL-12G group with ~12 Gb (three paired gastric cancer and tumor-adjacent samples of them were randomly selected) of paired-end reads generated per sample.

### Analyses of viral-related sequences in the transcriptome

For each sample, RNA-Seq data were trimmed using the Fastp program (33) with default parameters to remove adapter sequences and low-quality bases. After this step, clean reads were obtained, which were then aligned to the Homo Sapiens NCBI reference genome assembly version GRCh38 to filter out host-related reads using the HISAT2 (34) (v2.1.0). Subsequently, host-free reads were used for *de novo* assembly using Megahit (v1.2.9) and subjected to alignment with the non-redundant nucleotide databases and a more comprehensive database comprised of NCBI RefSeq viral reference genomes (18,729 sequences and phages) using BLASTn (v2.13.0) (35). Viral-related contigs with E-value <e^–5^ were retained. The clean reads were mapped back to each virus gene database from NCBI using HISAT2 (34), based on BAM files, removing unmapped viruses. Taxonomic information was obtained for the top blast hit and was assigned by TaxonKit (v0.2.4) (36). Finally, an index of 150 present reference virus genomes in single and whole virus databases was built using HISAT2-BUILD. Host-free reads and clean reads were, respectively, mapped back to those databases with HISAT2. Coverage and depth of viral genomes were calculated with Samtools (v1.9) (37) based on the SAM files. The determination of gene expression levels in viruses is also carried out using the above methods. To further enhance the quality of the genome annotations, we manually examined the BAM files of host-free reads and clean reads mapping to specific viral genomes using IGV (38). This process focused on removing errant alignment viruses and involved the following steps: ensuring alignment only to viral gene sequences; removing viruses with long single-repeating-base sequences; excluding viruses with multiple-repeating-base sequences; eliminating unreasonable viruses (e.g., plant and marine viruses); and removing low-abundance viruses found in few samples. After this screening process, we used NCBI’s VecScreen tool to exclude any carrier contamination.

Sequence similarity analysis of HERVs and host sequences was performed using HISAT2 and BLAST (39). The clean reads were mapped back to the HERVs database from NCBI using HISAT2, and then the HERV-related reads were aligned to the GRCh38 reference genome. Subsequently, the host genes were downloaded, and their positions were manually aligned and calibrated. The sequence similarity was then calculated using SimPlot (v3.5.1).

The normalized abundance (viral genomes per human diploid genome) of a virus in a gastric cancer sample was estimated with the following equation:


$$Virus\;abundance=75\;\times \;2\;\times \;10^{6}\;\times \frac{number\;of\;reads\;mapped\;to\;viral\;genome}{virus\;genome\;size\;\times \;total\;reads}$$

### RNA isolation, reverse transcription-PCR, and quantitative reverse transcription-PCR

The presence of viruses was further verified using RT-PCR or qRT-PCR with specific primer sequences targeting viral genes (Table S2). Total RNA was extracted from 10 paired gastric cancer and tumor-adjacent samples using TRIzol reagent (Invitrogen: Thermo Fisher Scientific, Carlsbad, CA, USA), reverse transcribed using a Rever Tra Ace qPCR RT Kit (Toyobo, Tokyo, Japan) according to the manufacturer’s instructions. cDNA was stored at −20°C until further use. The complementary DNA was then amplified to detect virus expression by qRT-PCR or PCR using a SYBR Green Master mix (QIAGEN) or the 2× Phanta Max Master Mix (Vazyme Biotech Co., Ltd., Nanjing, China) according to the manufacturer’s protocol. All tests were repeated independently three times. The mRNA expression was normalized to the expression of GAPDH mRNA and calculated using the 2^ΔCt^ method. PCR products were visualized on a 1% agarose gel stained with GelRed (Solarbio, Beijing, China), then sequenced by Sanger sequencing.

### Analyses of viral-related sequences in single-cell transcriptomes

The sequencing data were analyzed using CellRanger (version 7.0.1), and STAR (version 2.7.10b, https://github.com/alexdobin/STAR/releases/tag/2.7.10b) was applied to align reads to the genome GRCh38-2020-A and 64 reference virus whole genomes with high expression in gastric cancer. Seurat (version 4.4.0) (40) was employed to merge the expression matrices of all samples. There are two methods of data set quality control: performed and unperformed. Differentially expressed genes of each subset were identified using the FindAllMarkers function in Seurat. Cell type identification and annotation were performed based on differentially expressed genes within each cluster. Initially, automatic annotation of cell types was conducted using SingleR (v1.0.0) (41). Subsequently, manual annotation was carried out by referring to known marker genes of specific cell subtypes (Table S3) (42). This combined approach allowed for the accurate identification and annotation of cell types. The PercentageFeatureSet function in Seurat is designed to distinguish cells exhibiting viral expression from those expressing only host genes. Heatmaps were employed to visually represent the disparity in the quantity of virus-infected cells observed in samples subjected to quality control measures compared to those that were not.

### Microbial feature selection and construction of machine learning models

To identify a robust set of gastric cancer virome features for disease classification of discovery metatranscriptomic samples, we designed a parallel Random Forests model-based backward feature elimination (RF-BFE) algorithm guided by ranked feature importance scores (mean decrease in accuracy), with built-in hyper-parameter tuning through random search using the R implementation of Breiman and Cutler’s Random Forests algorithm. To evaluate the performance of the random forest model, we divided the data set into training and validation sets. Specifically, 70% of the samples were randomly selected to form the training set, which was used to train the model. The remaining 30% of the samples were used as the validation set to assess the model’s performance. In each complete round of backward feature elimination with a random permutation of mtry, ntree, and node size parameters, we computed stepwise average area under the curve (AUC) with balanced class weights and feature importance scores over 10 times repeated 10-fold cross-validations of the RF-BFE model (RF-BFE-AUC). The validity of the model can be judged by the AUC value. The significance of the area under the receiver operating characteristic curve under the null hypothesis of random predictive value was evaluated using the ROCR package in R.

### Statistical analysis

To evaluate the differences in viral expression levels across various groups, we employed the Kruskal-Wallis rank sum test. The groups included GC patients and tumor-adjacent, histopathological subtypes of gastric cancer, and *H. pylori* positive and negative cases. The statistical analysis and result visualization were performed using SPSS Statistics (version 23.0; IBM SPSS, Chicago, IL), GraphPad Prism 7 (GraphPad Software, San Diego, CA, USA), and RStudio (RStudio, Inc., Boston, MA, USA). Statistical significance was set at *P < 0.05, **P < 0.01, ***P < 0.001.

## Data Availability

The data from the article have been uploaded to GSA for Human under BioProject PRJCA042203 (HRA012113). scRNA-seq data files are available at the NCBI Sequence Read Archive (SRA) (https://www.ncbi.nlm.nih.gov/sra/) under BioProject accession PRJNA776683, GEO under accession GSE150290 (Bioproject: PRJNA631728; SRA: SRP261119), GSE163558 (Bioproject: PRJNA689571; SRA: SRP300226), and the Genome Sequence Archive (GSA) (https://ngdc.cncb.ac.cn/gsa/, data numbers PRJCA002596, HRA000051 and HRA003647).

## SUPPLEMENTAL MATERIAL

Supplemental material mbio.00551-25-s0001.pdf Figures S1-S6; Tables S1-S4.

ASM does not own the copyrights to Supplemental Material that may be linked to, or accessed through, an article. The authors have granted ASM a non-exclusive, world-wide license to publish the Supplemental Material files. Please contact the corresponding author directly for reuse.
