# Supplementary material for: Deciphering the viral landscape in gastric cancer: comprehensive characterization and identification of the gastric cancer virome
Source: mBio. 2025 Jul 9;16(8):e00551-25. doi: 10.1128/mbio.00551-25 (PMC12345221; doi:10.1128/mbio.00551-25)
Supplement: Supplemental material — Figures S1-S6; Tables S1-S4. [file mbio.00551-25-s0001.pdf]

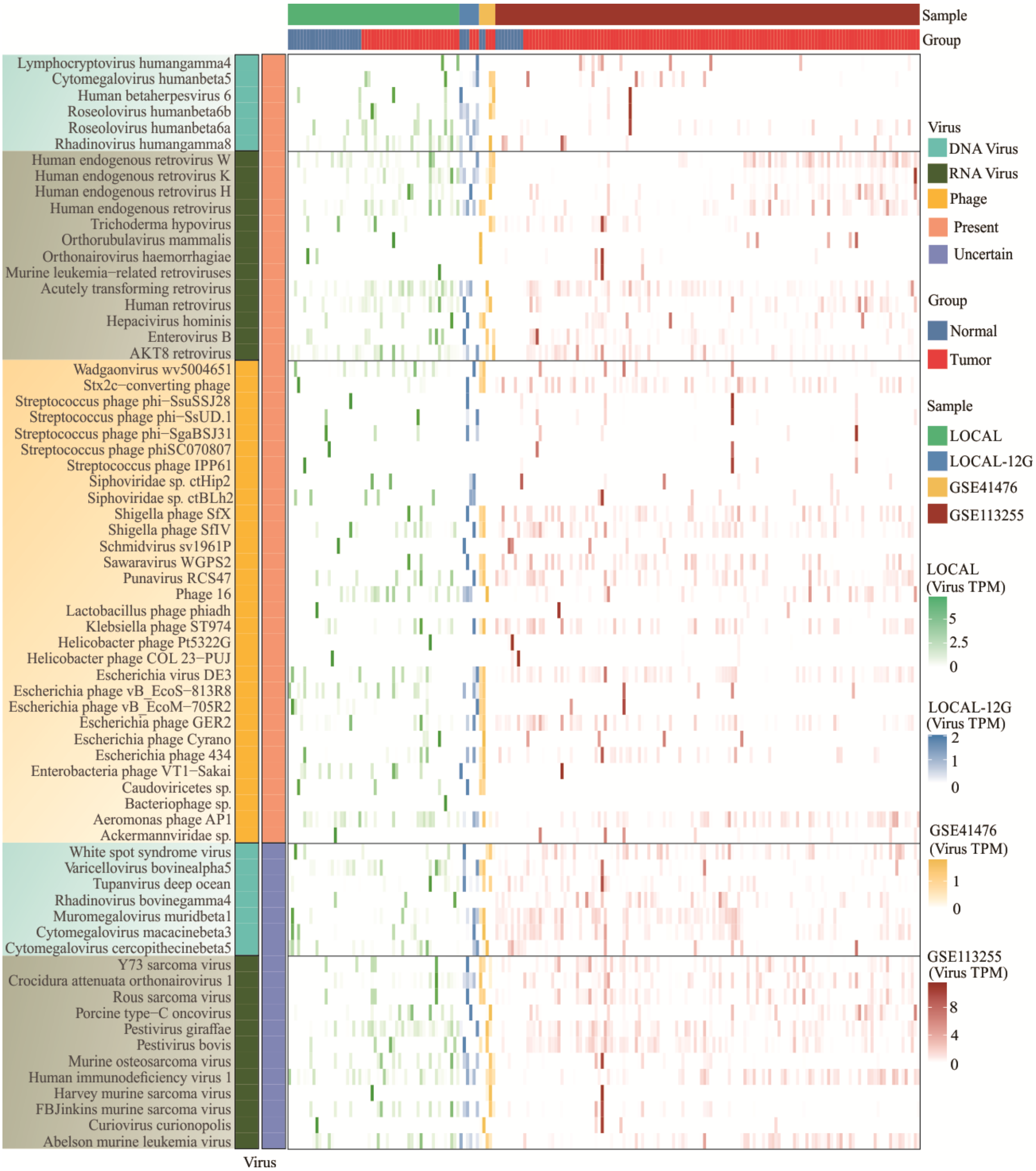

Supplementary Figure1 Overall view of the viral map in four GC transcriptome datasets. LOCAL-12G dataset includes tumor-adjacent (n=3 cases) and gastric cancer (n=3 cases), GSE41476 has tumor-adjacent (n=2 cases) and gastric cancer (n=3 cases).

A Present virus (15)

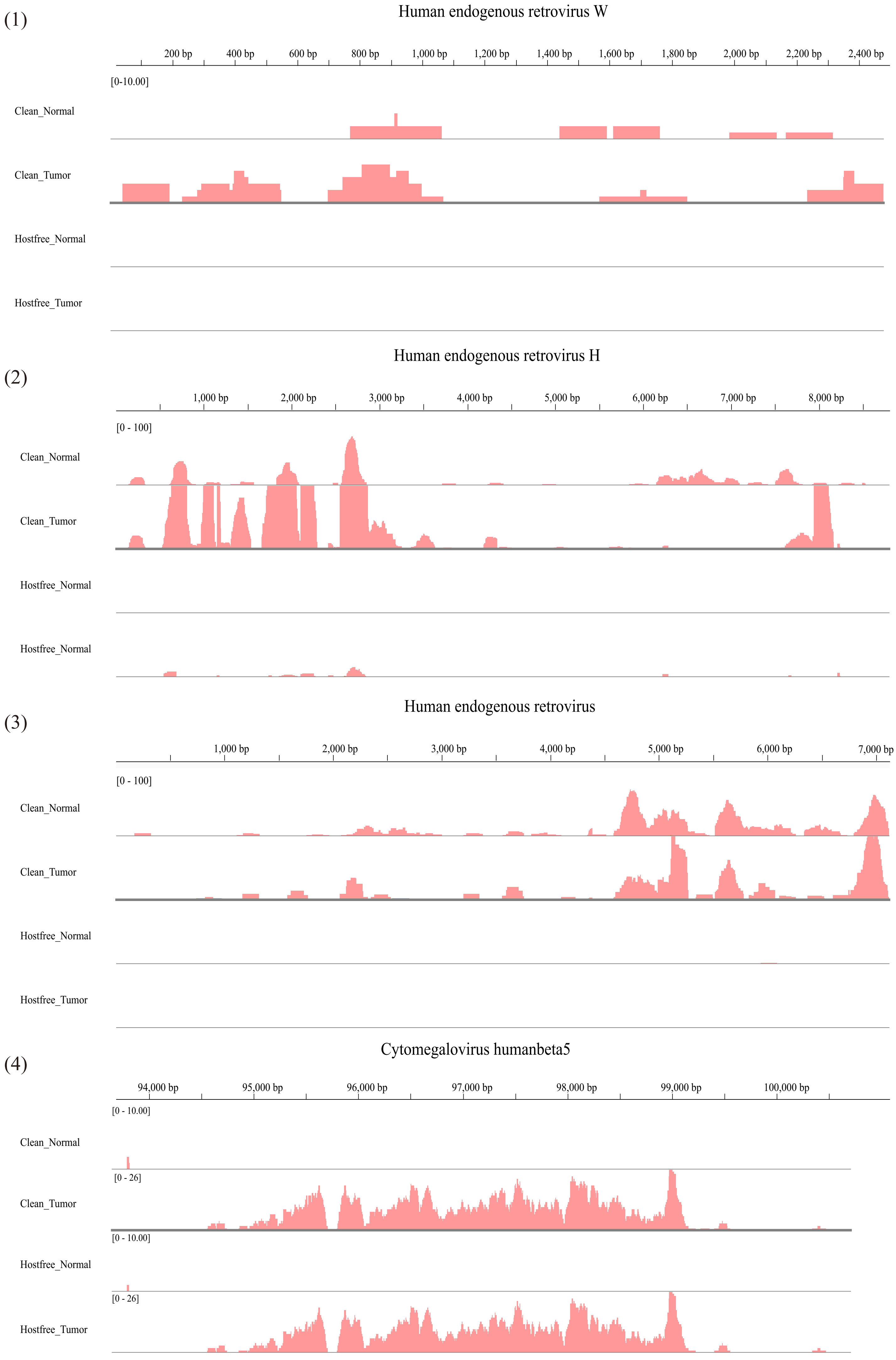

(5)

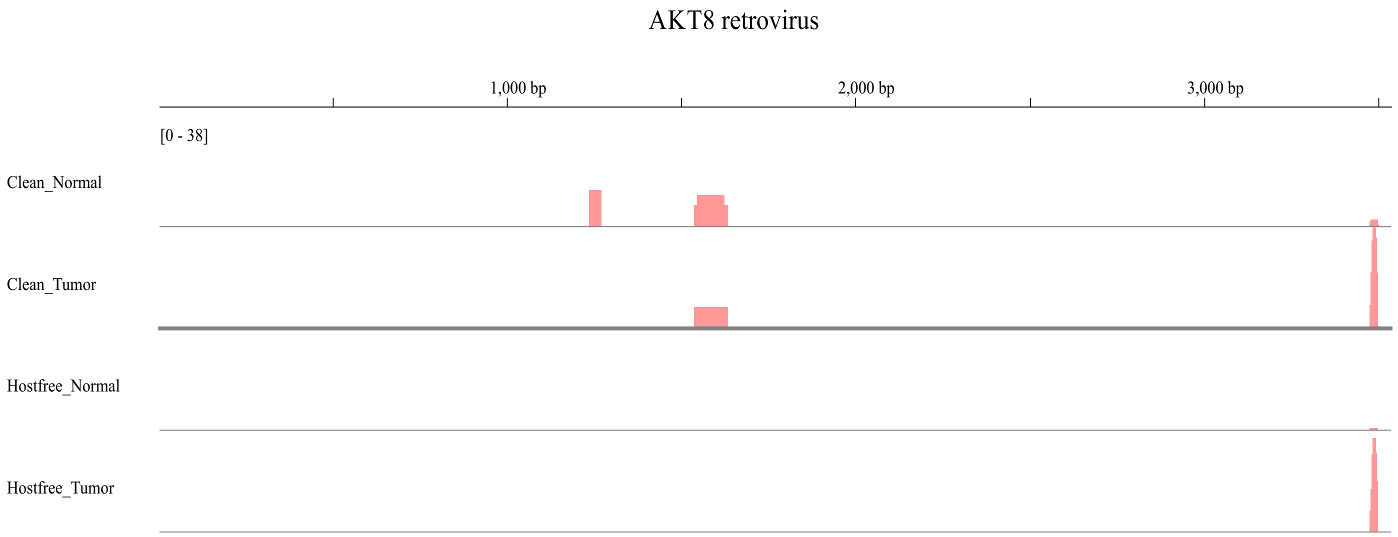

AKT8 retrovirus
1,000 bp
2,000 bp
3,000 bp
[0 - 38]
Clean_Normal
Clean_Tumor
Hostfree_Normal
Hostfree_Tumor

(6)

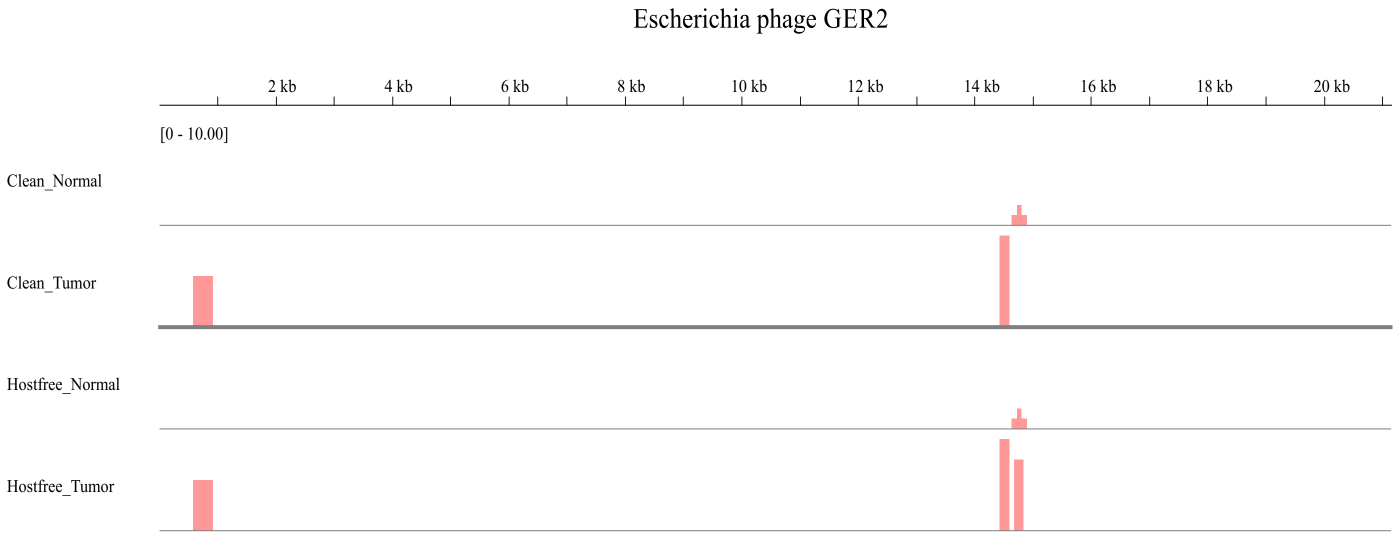

Escherichia phage GER2
2 kb
4 kb
6 kb
8 kb
10 kb
12 kb
14 kb
16 kb
18 kb
20 kb
[0 - 10.00]
Clean_Normal
Clean_Tumor
Hostfree_Normal
Hostfree_Tumor

(7)

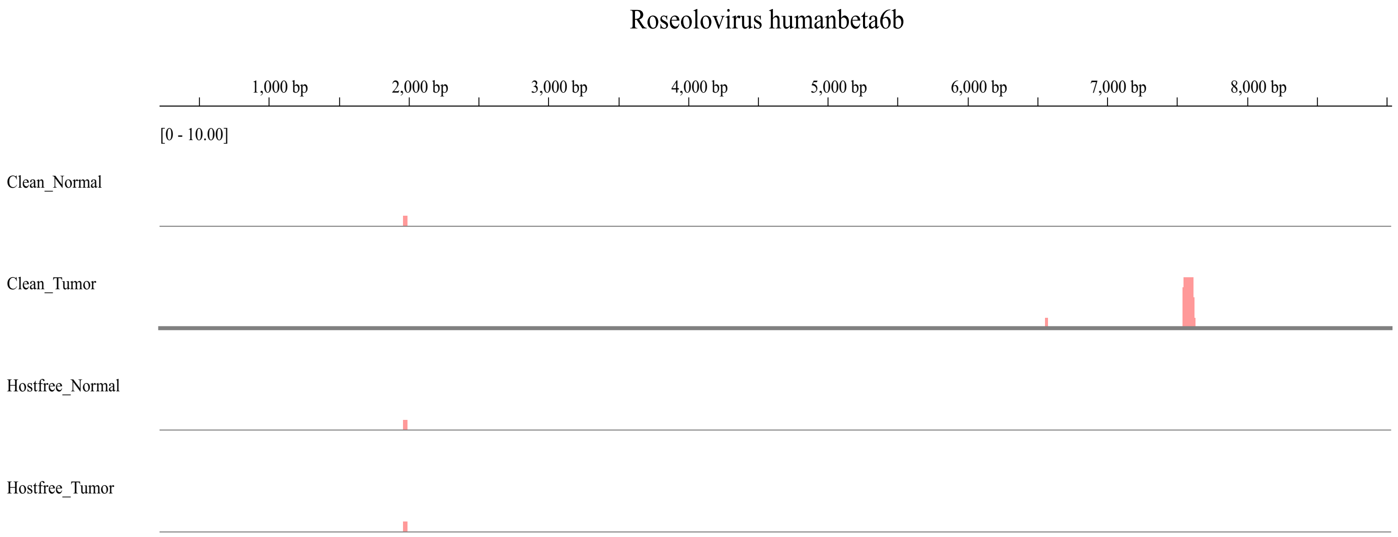

Roseolovirus humanbeta6b
1,000 bp
2,000 bp
3,000 bp
4,000 bp
5,000 bp
6,000 bp
7,000 bp
8,000 bp
[0 - 10.00]
Clean_Normal
Clean_Tumor
Hostfree_Normal
Hostfree_Tumor

(8)

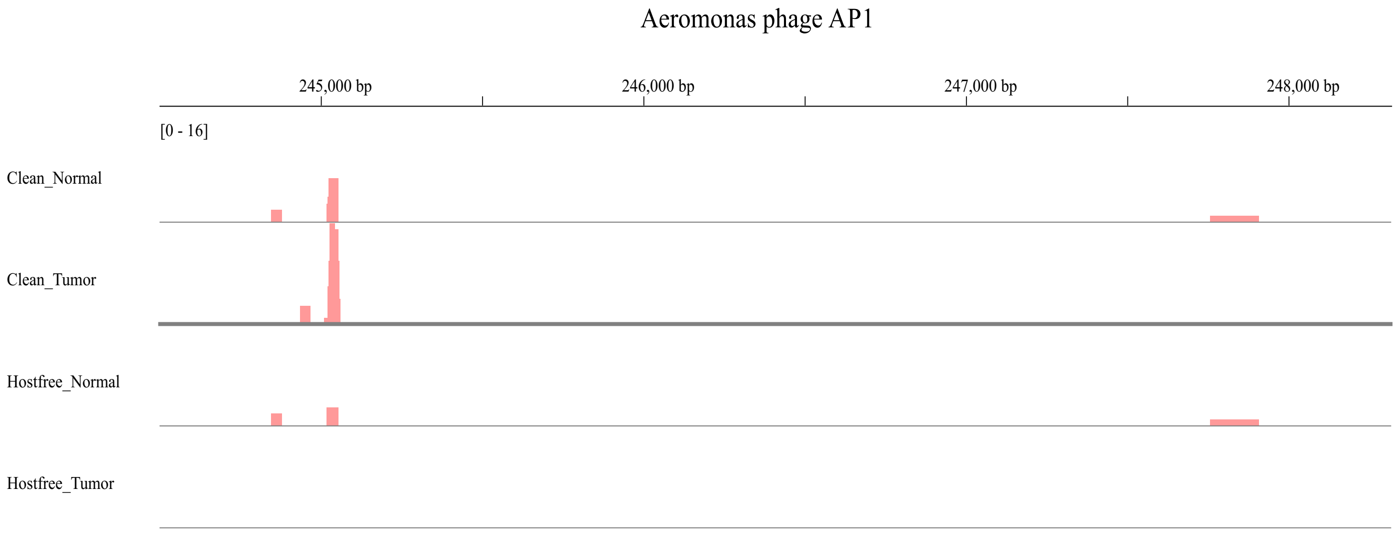

Aeromonas phage AP1
245,000 bp
246,000 bp
247,000 bp
248,000 bp
[0 - 16]
Clean_Normal
Clean_Tumor
Hostfree_Normal
Hostfree_Tumor

(9)

Roseolovirus humanbeta6a

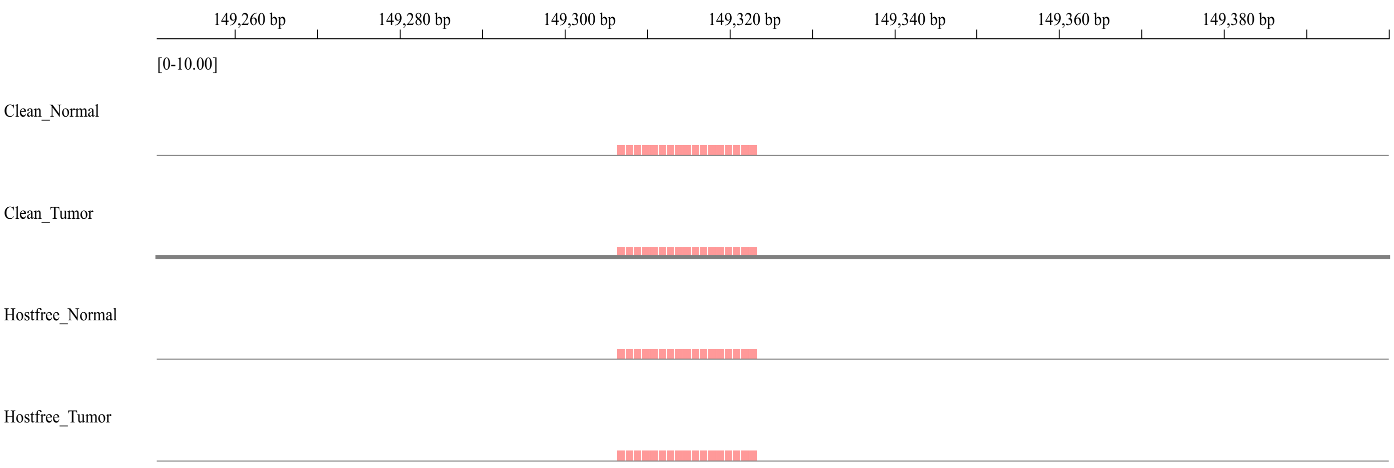

(10)

Rhadinovirus humangamma8

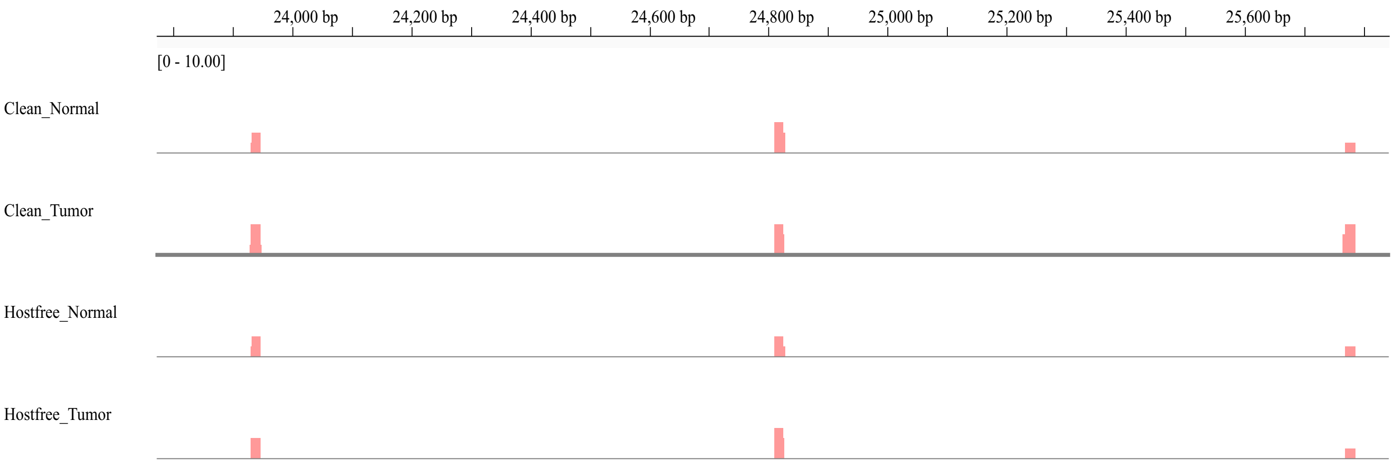

(11)

Shigella phage SfIV

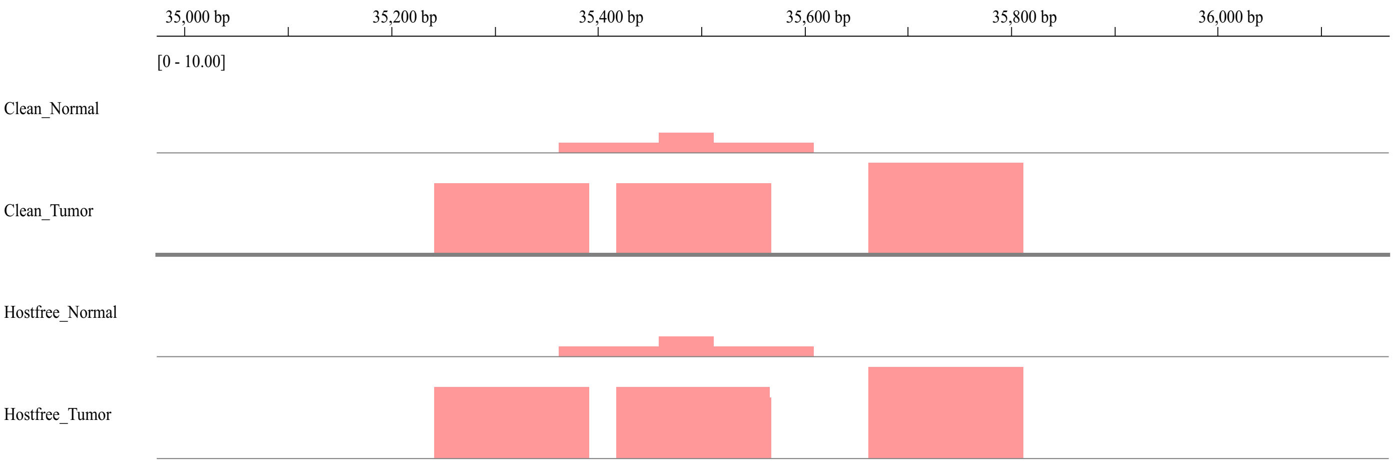

(12)

Escherichia virus DE3

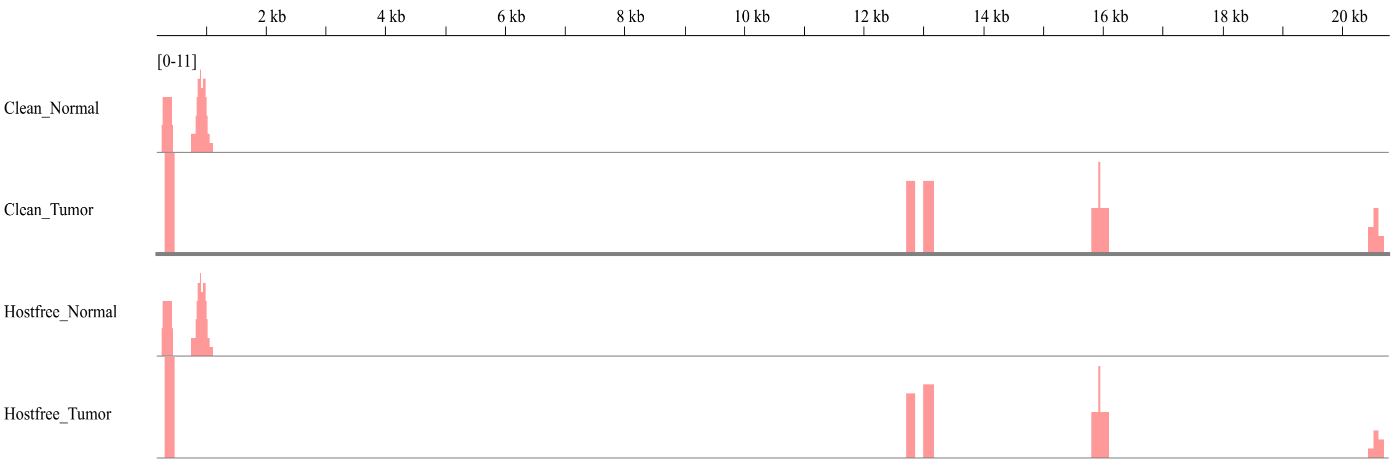

(13)

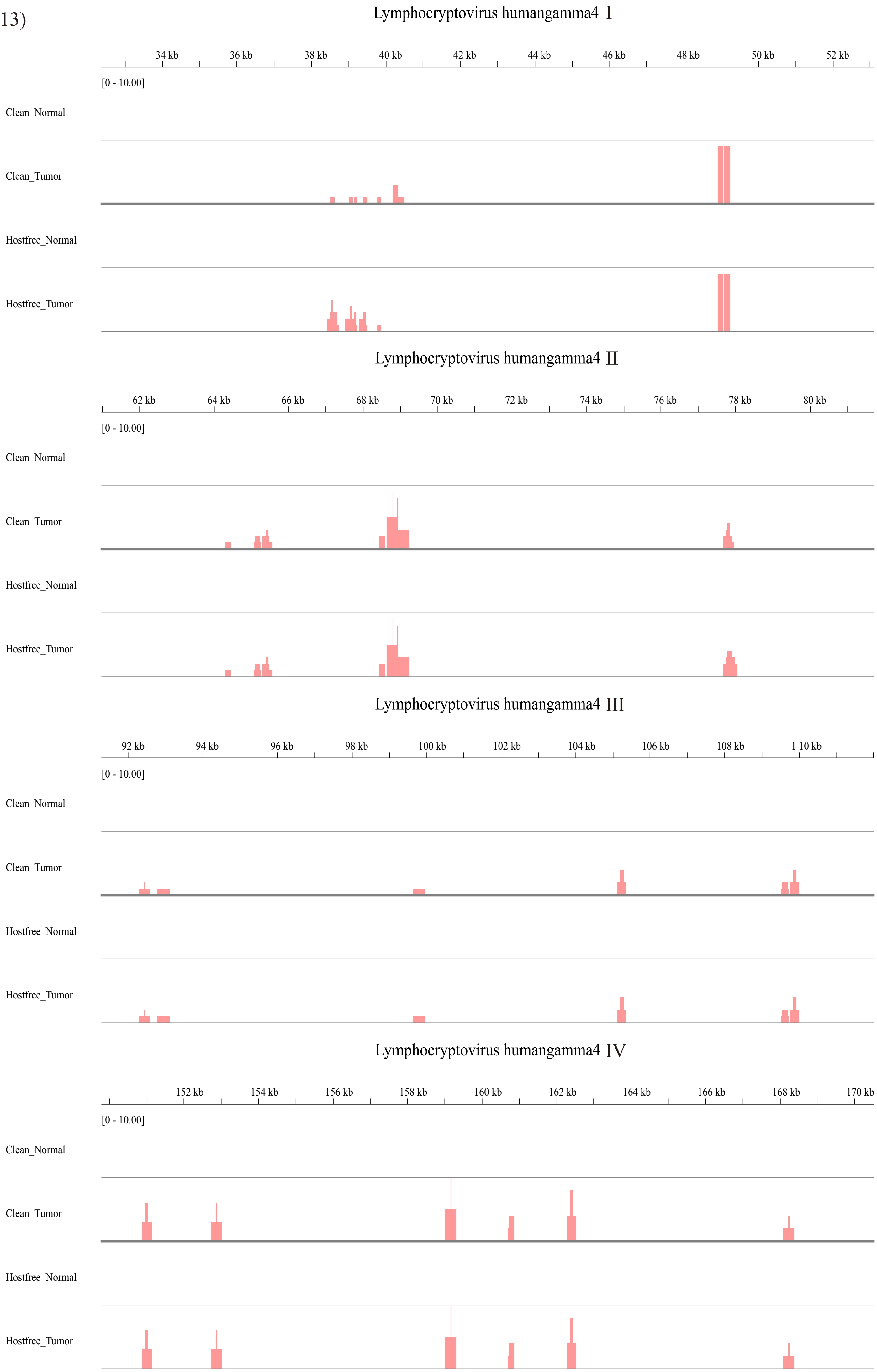

Lymphocryptovirus humangamma4 I
34 kb
36 kb
38 kb
40 kb
42 kb
44 kb
46 kb
48 kb
50 kb
52 kb
[0 - 10.00]
Clean_Normal
Clean_Tumor
Hostfree_Normal
Hostfree_Tumor
Lymphocryptovirus humangamma4 II
62 kb
64 kb
66 kb
68 kb
70 kb
72 kb
74 kb
76 kb
78 kb
80 kb
[0 - 10.00]
Clean_Normal
Clean_Tumor
Hostfree_Normal
Hostfree_Tumor
Lymphocryptovirus humangamma4 III
92 kb
94 kb
96 kb
98 kb
100 kb
102 kb
104 kb
106 kb
108 kb
1 10 kb
[0 - 10.00]
Clean_Normal
Clean_Tumor
Hostfree_Normal
Hostfree_Tumor
Lymphocryptovirus humangamma4 IV
152 kb
154 kb
156 kb
158 kb
160 kb
162 kb
164 kb
166 kb
168 kb
170 kb
[0 - 10.00]
Clean_Normal
Clean_Tumor
Hostfree_Normal
Hostfree_Tumor

(14)

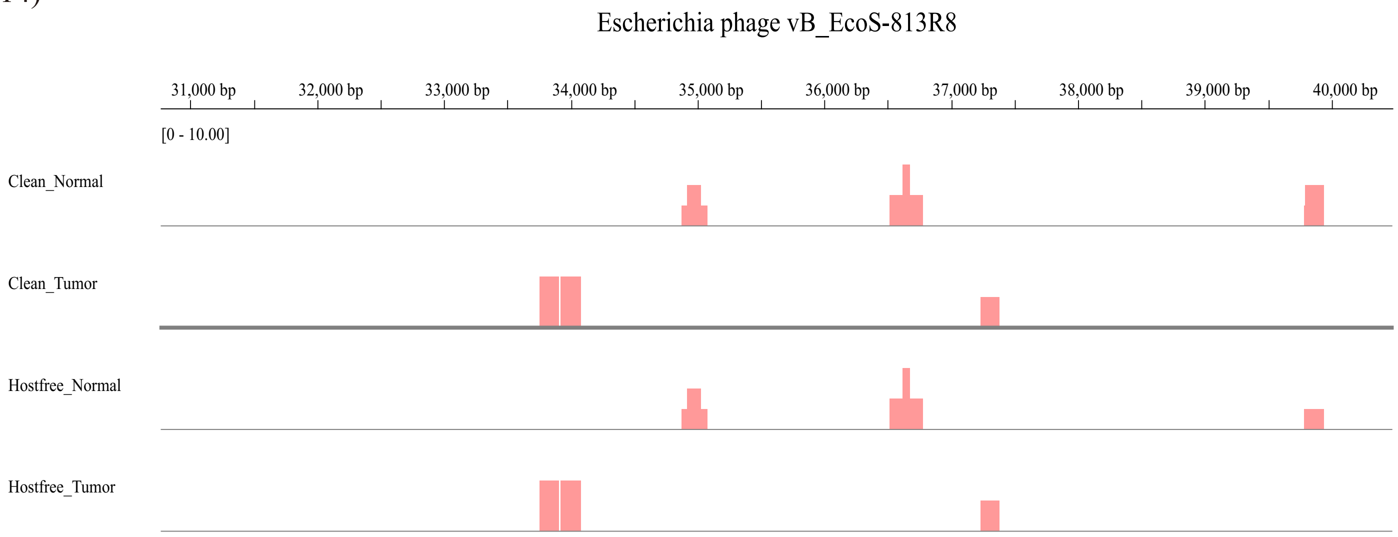
Escherichia phage vB_EcoS-813R8
31,000 bp
32,000 bp
33,000 bp
34,000 bp
35,000 bp
36,000 bp
37,000 bp
38,000 bp
39,000 bp
40,000 bp
[0 - 10.00]
Clean_Normal
Clean_Tumor
Hostfree_Normal
Hostfree_Tumor

(15)

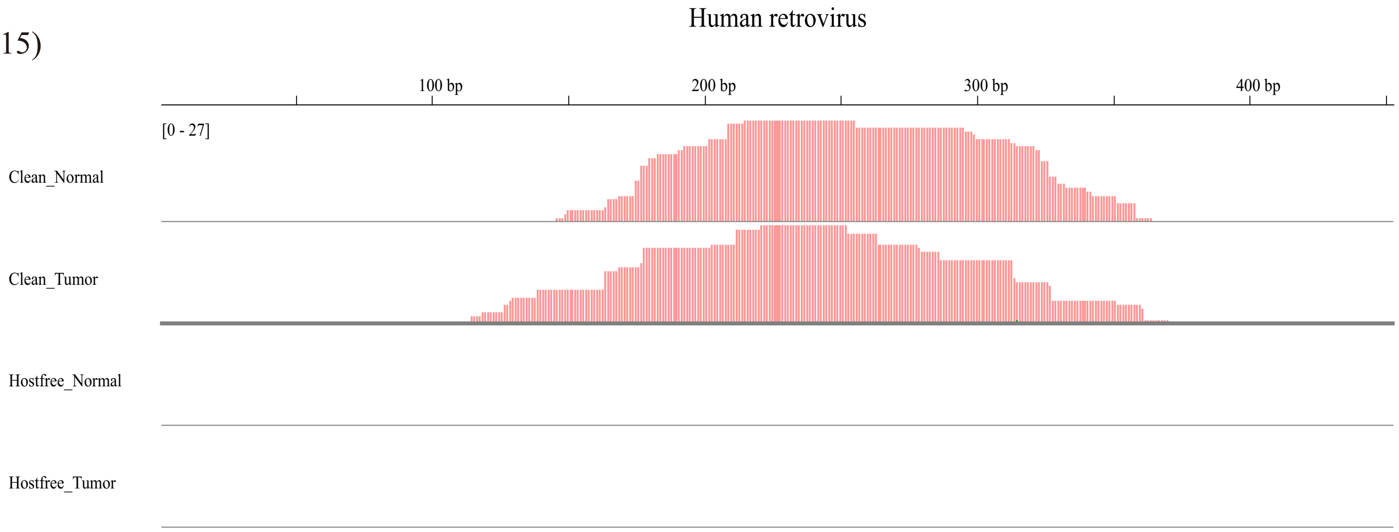
Human retrovirus
100 bp
200 bp
300 bp
400 bp
[0 - 27]
Clean_Normal
Clean_Tumor
Hostfree_Normal
Hostfree_Tumor

B Uncertain virus (11)

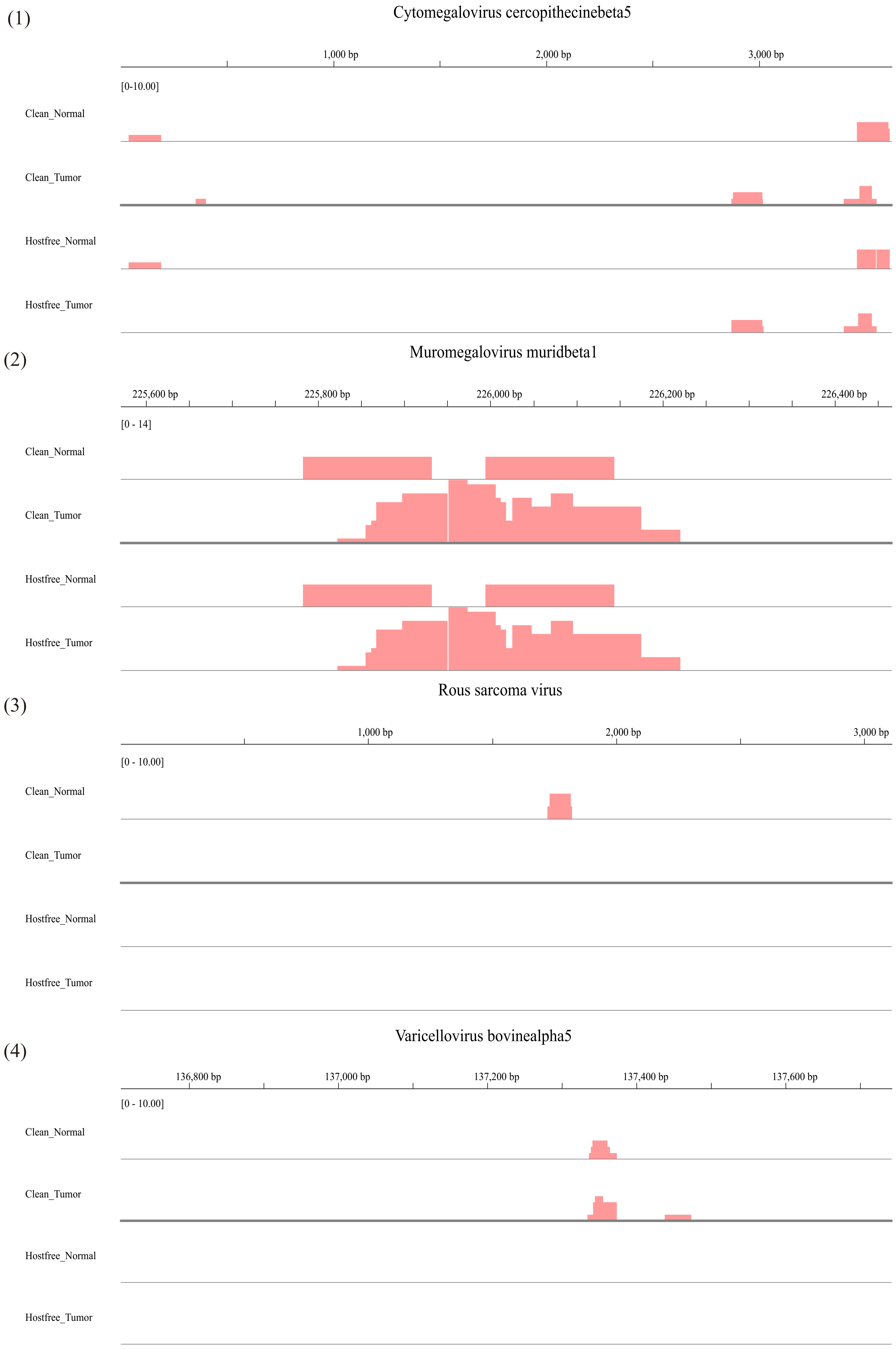

(5)

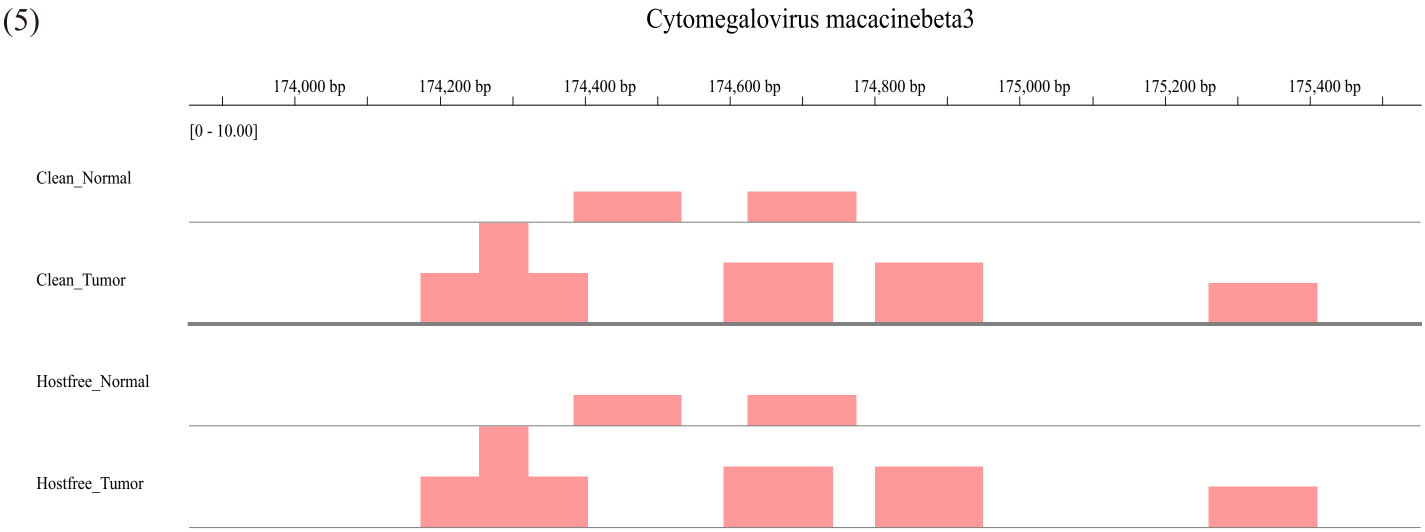

Cytomegalovirus macacinebeta3
174,000 bp
174,200 bp
174,400 bp
174,600 bp
174,800 bp
175,000 bp
175,200 bp
175,400 bp
[0 - 10.00]
Clean_Normal
Clean_Tumor
Hostfree_Normal
Hostfree_Tumor

(6)

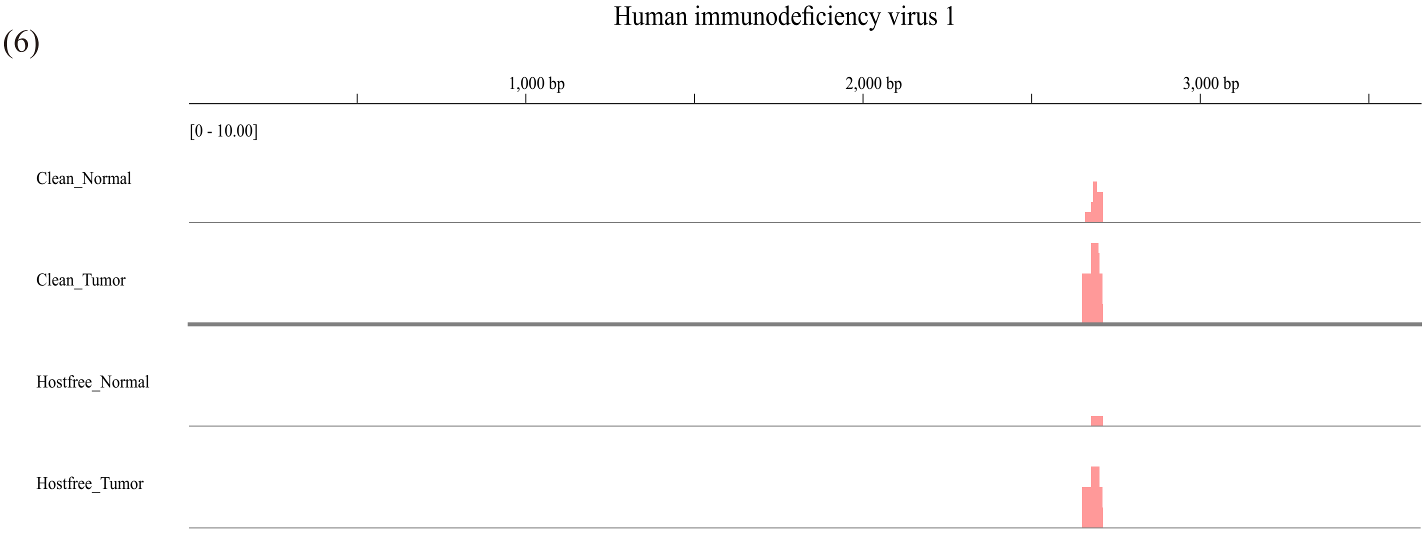

Human immunodeficiency virus 1
1,000 bp
2,000 bp
3,000 bp
[0 - 10.00]
Clean_Normal
Clean_Tumor
Hostfree_Normal
Hostfree_Tumor

(7)

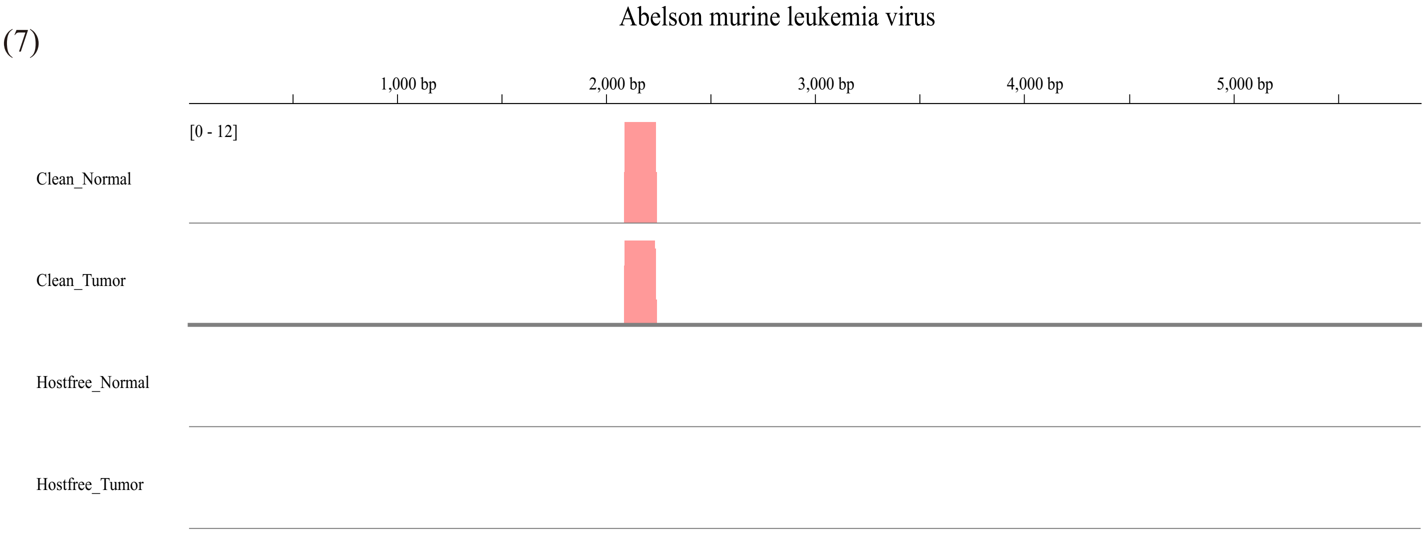

Abelson murine leukemia virus
1,000 bp
2,000 bp
3,000 bp
4,000 bp
5,000 bp
[0 - 12]
Clean_Normal
Clean_Tumor
Hostfree_Normal
Hostfree_Tumor

(8)

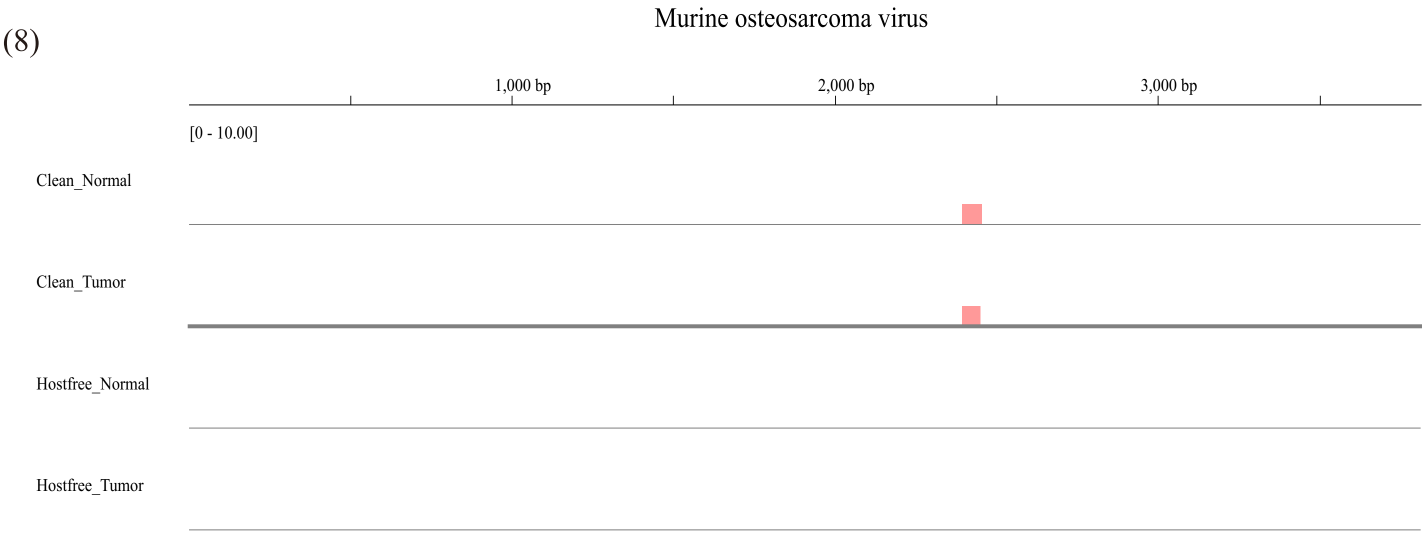

Murine osteosarcoma virus
1,000 bp
2,000 bp
3,000 bp
[0 - 10.00]
Clean_Normal
Clean_Tumor
Hostfree_Normal
Hostfree_Tumor

(9)

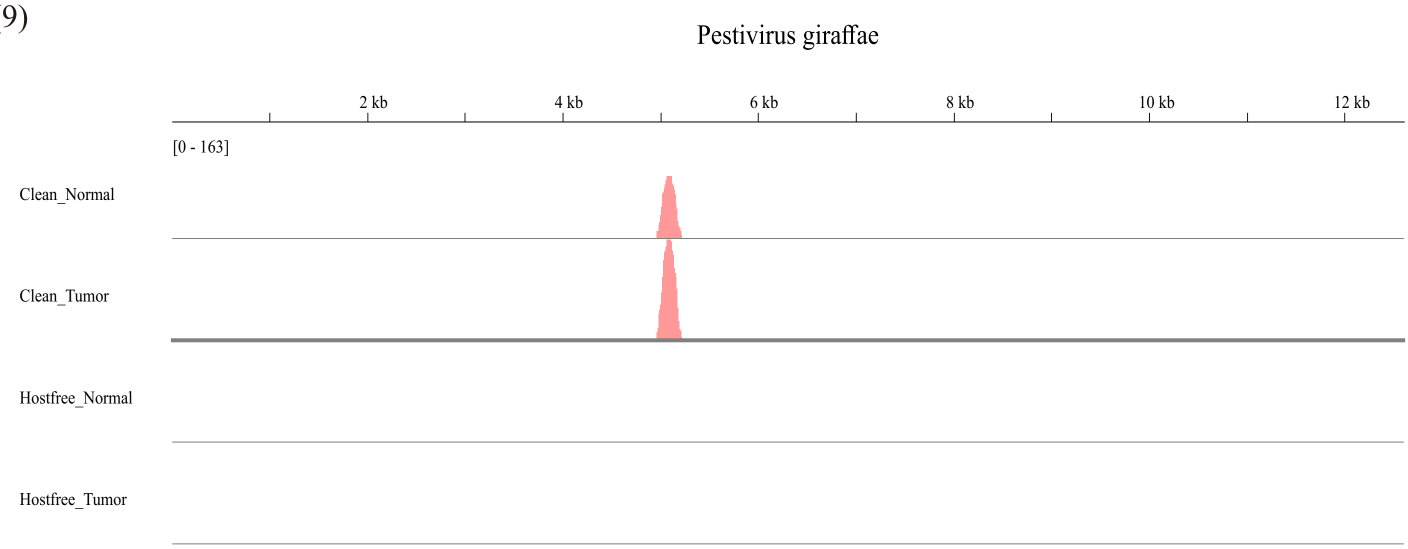
Pestivirus giraffae
2 kb
4 kb
6 kb
8 kb
10 kb
12 kb
[0 - 163]
Clean_Normal
Clean_Tumor
Hostfree_Normal
Hostfree_Tumor

(10)

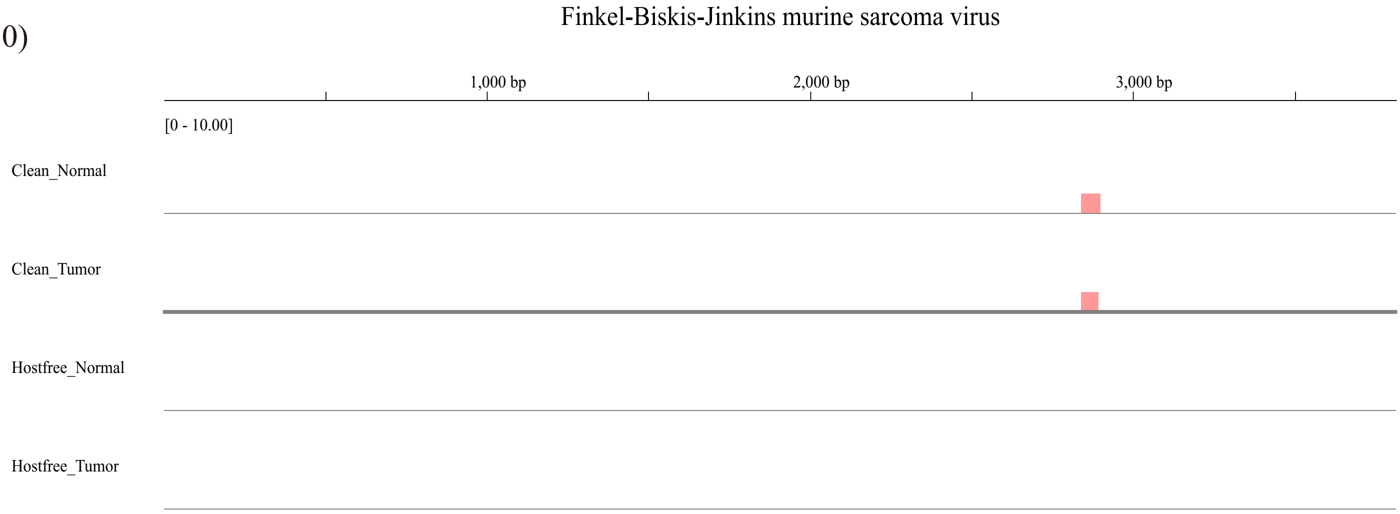
Finkel-Biskis-Jinkins murine sarcoma virus
1,000 bp
2,000 bp
3,000 bp
[0 - 10.00]
Clean_Normal
Clean_Tumor
Hostfree_Normal
Hostfree_Tumor

(11)

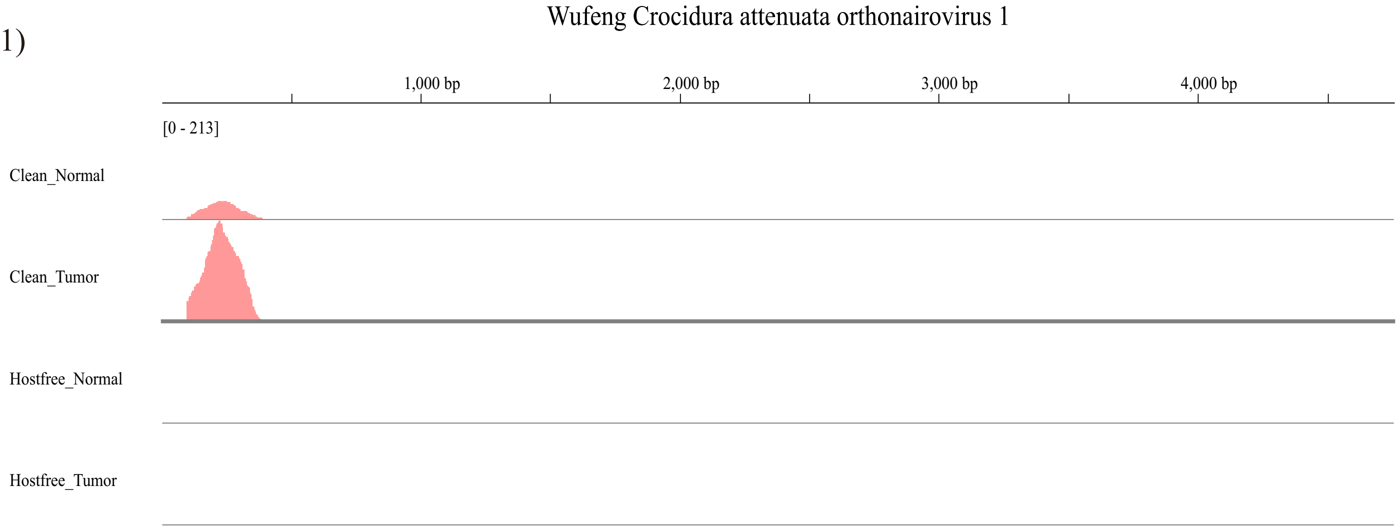
Wufeng Crocidura attenuata orthonairovirus 1
1,000 bp
2,000 bp
3,000 bp
4,000 bp
[0 - 213]
Clean_Normal
Clean_Tumor
Hostfree_Normal
Hostfree_Tumor

Supplementary Figure2 IGV visualizes the expression of viral genome. (A) 15 Present viruses, (B) 11 Uncertain viruses.

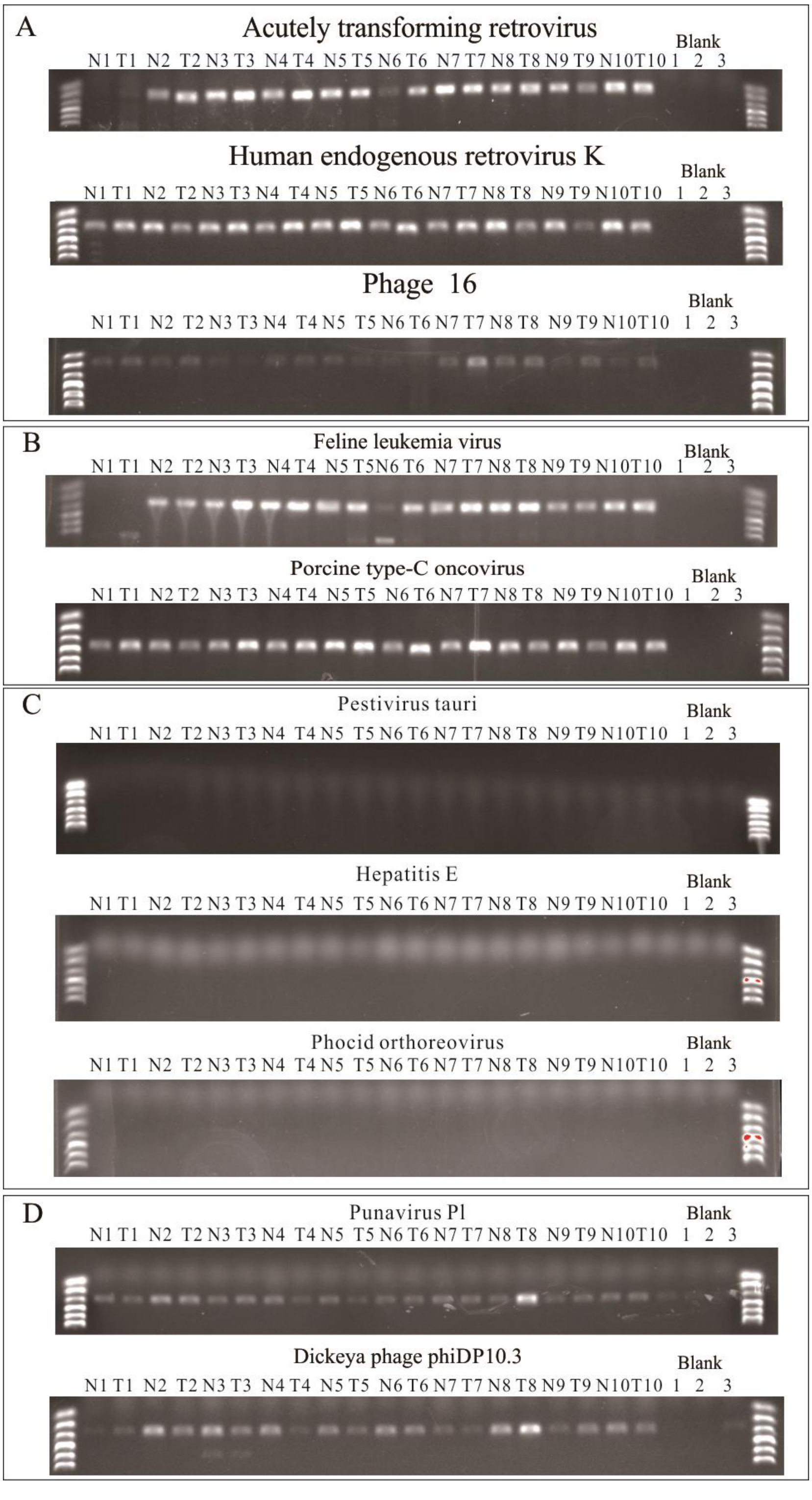

Supplementary Figure3 The results of RT-PCR products after agarose gel electrophoresis. The numbers below the lanes represent the code for a particular patient, with "T" for tumor tissue and "N" for the corresponding adjacent normal gastric tissues. (A) Three virus were confirmed by RT-PCR, (B) Feline leukemia virus and Porcine type-C oncovirus are known to be potential sources of contamination in sequencing and alignment processes, (C) The PCR results for three viruses did not show any bands, (D) both the tissues and blank controls of Punavirus P1 and Dickeya phage phiDP10.3 displayed segments.

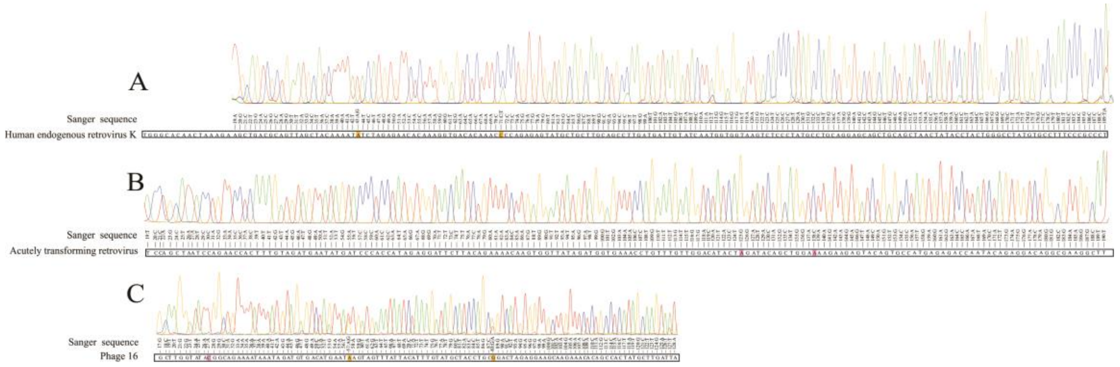

Supplementary Figure4 Sequencing detects these three viruses sequences. The sequencing results of three viruses show good homology with the target fragment.

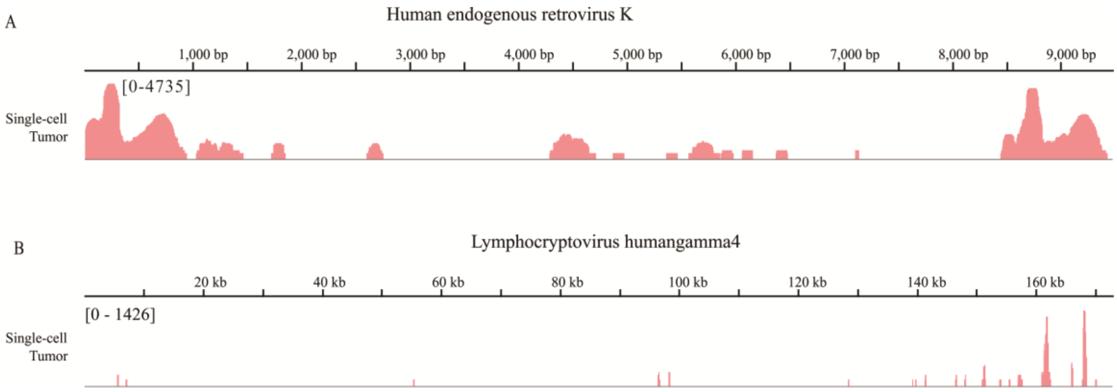

Supplementary Figure5 IGV visualizes the expression of HERV-K and Lymphocryptovirus humangamma4.

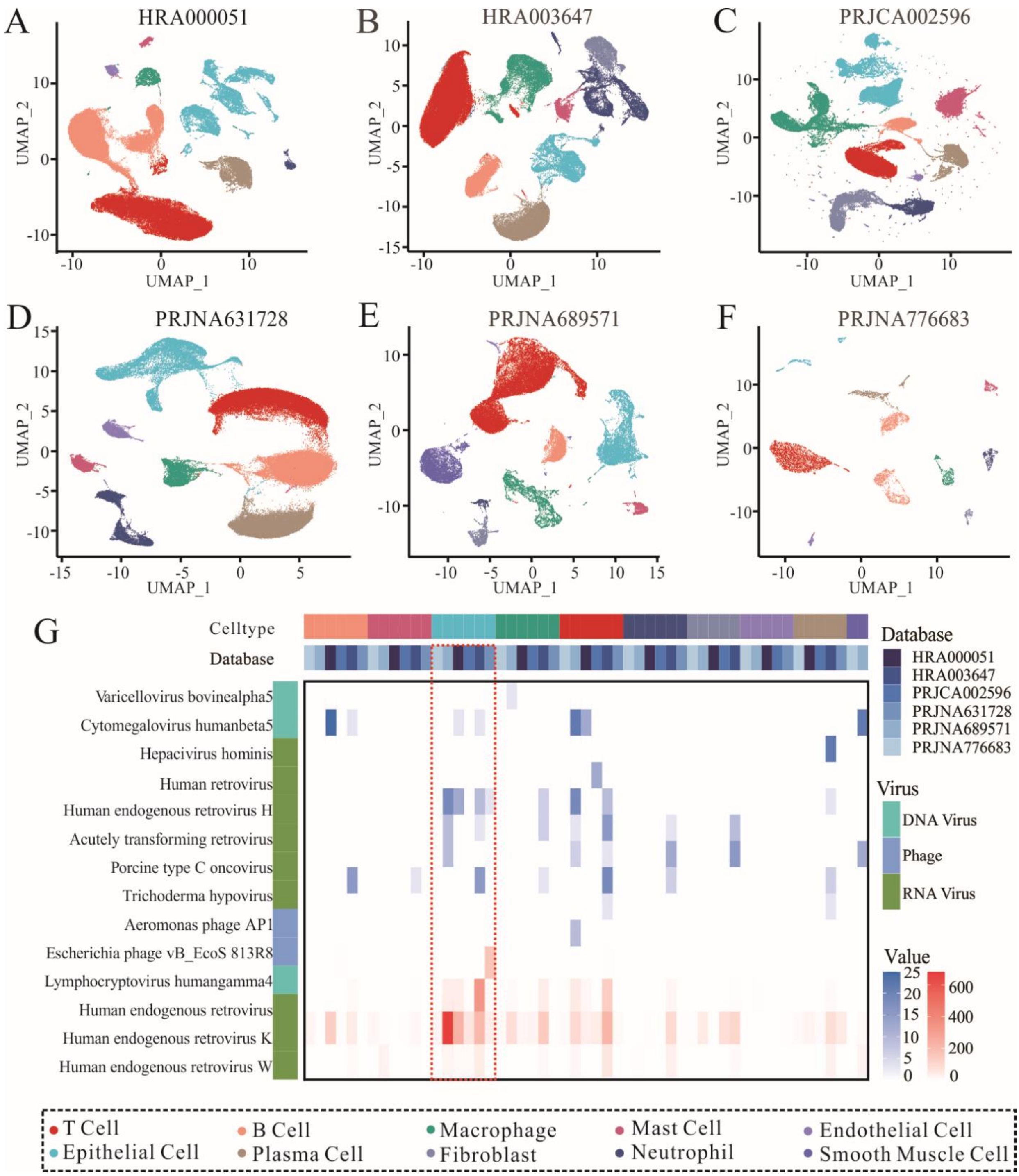

Supplementary Figure6 Single-cell transcriptome show the celltype and number of cells in which the virus is present after quality control. (A-F) Umap dimensionality reduction and manual annotation of six single-cell transcriptome datasets after quality control. (G) The heatmaps based on celltype and number of cells in which the virus is present.

Supplementary Table 1 Clinical information

Num	Age	Gender	Stage	Differentiation	Lauren	Hp	Tissue
78T	58	Female	III	Low	Mixed	Positive	gastric cancer
81T	84	Male	I	Low	Intestinal	Negative	gastric cancer
82T	73	Male	II	Medium-Low	Mixed	Negative	gastric cancer
84T	71	Male	III	High-Medium	Mixed	Negative	gastric cancer
85T	85	Female	III	Medium-Low	Mixed	Negative	gastric cancer
90T	61	Female	III	Low	Diffusedd	Negative	gastric cancer
94T	75	Female	III	Low-None	Intestinal	Negative	gastric cancer
96T	53	Female	III	Low	Mixed	Negative	gastric cancer
98T	88	Male	III	Low	Mixed	Negative	gastric cancer
99T	56	Male	II	Low	Mixed	Negative	gastric cancer
103T	69	Female	III	Medium-Low	Mixed	Negative	gastric cancer
106T	45	Male	III	High-Medium	Intestinal	Negative	gastric cancer
108T	72	Female	II	Medium	Intestinal	Negative	gastric cancer
109T	72	Female	II	Medium	Intestinal	Negative	gastric cancer
110T	41	Male	III	Low	Diffusedd	Negative	gastric cancer
111T	79	Male	III	Low	Mixed	Negative	gastric cancer
112T	72	Female	III	Low	Mixed	Positive	gastric cancer
113T	68	Female	I	Medium	Intestinal	Positive	gastric cancer
116T	65	Male	II	Medium	Intestinal	Negative	gastric cancer
117T	79	Female	I	Low	Diffusedd	Positive	gastric cancer
119T	51	Female	III	Low	Diffusedd	Positive	gastric cancer
120T	64	Female	I	Medium	Intestinal	Negative	gastric cancer
121T	59	Female	II	Medium	Intestinal	Negative	gastric cancer
122T	64	Male	III	Medium	Intestinal	Negative	gastric cancer
124T	72	Female	III	Low	Diffusedd	Negative	gastric cancer
125T	91	Female	III	Medium-Low	Mixed	Positive	gastric cancer
126T	71	Female	I	High	Mixed	Negative	gastric cancer
127T	58	Male	III	Medium-Low	Mixed	Negative	gastric cancer
128T	69	Male	III	Low	Mixed	Negative	gastric cancer
129T	73	Female	III	Medium-Low	Mixed	Negative	gastric cancer
130T	72	Female	III	High-Medium	Intestinal	Negative	gastric cancer
131T	42	Male	II	Low	Diffusedd	Negative	gastric cancer
SRR585571						Positive	normal
SRR585575						Positive	gastric cancer
SRR585576						Negative	gastric cancer
SRR585577						Negative	gastric cancer
SRR585570						Negative	normal
SRR7014291	53	Female			Intestinal	Positive	gastric cancer
SRR7014292	81	Male			Diffused	Negative	gastric cancer
SRR7014293	89	Female			Diffused	Negative	gastric cancer
SRR7014294	44	Male			Diffused	Positive	gastric cancer
SRR7014295	81	Female			Intestinal	Positive	gastric cancer
SRR7014296	86	Female			Intestinal	Positive	gastric cancer
SRR7014297	48	Female			Diffused	Negative	gastric cancer
SRR7014298						Positive	normal
SRR7014299	74	Male			Intestinal	Positive	gastric cancer
SRR7014300						Negative	normal
SRR7014301	81	Male			Diffused	Negative	gastric cancer
SRR7014302						Positive	normal

SRR7014303	70	Female			Intestinal	Positive	gastric cancer
SRR7014304	58	Male			Diffused	Positive	gastric cancer
SRR7014305	65	Female			Diffused	Positive	gastric cancer
SRR7014306	78	Female			Diffused	Positive	gastric cancer
SRR7014307	79	Male			Diffused	Negative	gastric cancer
SRR7014308						Negative	normal
SRR7014309	73	Female			Intestinal	Positive	gastric cancer
SRR7014310	56	Male			Diffused	Negative	gastric cancer
SRR7014311	52	Female			Diffused	Positive	gastric cancer
SRR7014312	71	Male			Intestinal	Positive	gastric cancer
SRR7014313	61	Male			Intestinal	Positive	gastric cancer
SRR7014314	52	Male			Intestinal	Negative	gastric cancer
SRR7014315	46	Male			Diffused	Positive	gastric cancer
SRR7014316	74	Male			Diffused	Positive	gastric cancer
SRR7014317	52	Male			Diffused	Positive	gastric cancer
SRR7014318	80	Female			Diffused	Negative	gastric cancer
SRR7014319	55	Male			Diffused	Positive	gastric cancer
SRR7014320							normal
SRR7014321	65	Female			Intestinal	Positive	gastric cancer
SRR7014322	57	Male			Diffused	Positive	gastric cancer
SRR7014323	54	Male			Diffused	Negative	gastric cancer
SRR7014324	63	Female			Diffused	Positive	gastric cancer
SRR7014325	51	Female			Diffused	Positive	gastric cancer
SRR7014326	68	Female			Diffused	Positive	gastric cancer
SRR7014327	55	Female			Diffused	Positive	gastric cancer
SRR7014328	72	Female			Diffused	Positive	gastric cancer
SRR7014329	65	Male			Diffused	Positive	gastric cancer
SRR7014330	64	Male			Diffused	Negative	gastric cancer
SRR7014331	44	Female			Diffused	Negative	gastric cancer
SRR7014332	67	Male			Diffused	Negative	gastric cancer
SRR7014333	46	Female			Diffused	Positive	gastric cancer
SRR7014334	34	Male			Diffused	Positive	gastric cancer
SRR7014335	56	Male			Diffused	Positive	gastric cancer
SRR7014336						Positive	normal
SRR7014337	75	Male			Intestinal	Positive	gastric cancer
SRR7014338	42	Male			Diffused	Negative	gastric cancer
SRR7014339	75	Male			Diffused	Positive	gastric cancer
SRR7014340	55	Male			Diffused	Positive	gastric cancer
SRR7014341	44	Male			Diffused	Positive	gastric cancer
SRR7014342	68	Female			Diffused	Positive	gastric cancer
SRR7014343	56	Female			Diffused	Positive	gastric cancer
SRR7014344	48	Female			Diffused	Positive	gastric cancer
SRR7014345	56	Female			Diffused	Negative	gastric cancer
SRR7014346	67	Male			Diffused	Negative	gastric cancer
SRR7014347	25	Female			Diffused	Positive	gastric cancer
SRR7014348						Positive	normal
SRR7014349	66	Male			Intestinal	Positive	gastric cancer
SRR7014350						Positive	normal
SRR7014351	80	Male			Intestinal	Positive	gastric cancer
SRR7014352	60	Male			Intestinal	Positive	gastric cancer
SRR7014353	60	Male			Intestinal	Negative	gastric cancer
SRR7014354	73	Female			Intestinal	Positive	gastric cancer
SRR7014355	66	Male			Diffused	Positive	gastric cancer

SRR7014356	70	Male			Diffused	Positive	gastric cancer
SRR7014357	59	Male			Diffused	Positive	gastric cancer
SRR7014358	59	Male			Diffused	Positive	gastric cancer
SRR7014359						Positive	normal
SRR7014360	59	Male			Intestinal	Positive	gastric cancer
SRR7014361	77	Male			Diffused	Positive	gastric cancer
SRR7014362	60	Male			Intestinal	Positive	gastric cancer
SRR7014363	52	Male			Intestinal	Negative	gastric cancer
SRR7014364						Positive	normal
SRR7014365	44	Female			Intestinal	Positive	gastric cancer
SRR7014366	68	Female			Diffused	Positive	gastric cancer
SRR7014367	72	Female			Diffused	Positive	gastric cancer
SRR7014368	81	Female			Intestinal	Positive	gastric cancer
SRR7014369	35	Male			Diffused	Positive	gastric cancer
SRR7014370	77	Male			Intestinal	Positive	gastric cancer
SRR7014371	80	Male			Intestinal	Positive	gastric cancer
SRR7014372	59	Male			Diffused	Positive	gastric cancer
SRR7014373	80	Male			Diffused	Positive	gastric cancer
SRR7014374	75	Female			Diffused	Positive	gastric cancer
SRR7014375	66	Male			Diffused	Positive	gastric cancer
SRR7014376	45	Male			Diffused	Positive	gastric cancer
SRR7014377	62	Male			Diffused	Positive	gastric cancer
SRR7014378	71	Female			Diffused	Positive	gastric cancer
SRR7014379	44	Female			Diffused	Positive	gastric cancer
SRR7014380	40	Male			Diffused	Positive	gastric cancer
SRR7014381	75	Male			Diffused	Positive	gastric cancer
SRR7014382	72	Female			Diffused	Negative	gastric cancer
SRR7014383	43	Male			Diffused	Negative	gastric cancer
SRR7014384	57	Male			Diffused	Negative	gastric cancer
SRR7014385	64	Male			Diffused	Positive	gastric cancer
SRR7014386	48	Female			Diffused	Positive	gastric cancer
SRR7014387	63	Female			Diffused	Positive	gastric cancer
SRR7014388	48	Male			Diffused	Positive	gastric cancer
SRR7014389	84	Male			Diffused	Positive	gastric cancer
SRR7014390	51	Male			Diffused	Positive	gastric cancer
SRR7014391	66	Male			Diffused	Negative	gastric cancer
SRR7014392	49	Male			Diffused	Positive	gastric cancer
SRR7014393	78	Male			Diffused		gastric cancer
SRR7014394	77	Female			Diffused	Positive	gastric cancer
SRR7014395	72	Male			Diffused	Positive	gastric cancer
SRR7014396	59	Female			Diffused	Positive	gastric cancer
SRR7014397	72	Male			Diffused		gastric cancer
SRR7014398	50	Male			Diffused	Positive	gastric cancer
SRR7014399	53	Female			Diffused	Positive	gastric cancer
SRR7014400	66	Female			Diffused	Positive	gastric cancer
SRR7014401	56	Male			Diffused	Positive	gastric cancer
SRR7014402	78	Female			Diffused	Positive	gastric cancer
SRR7014403	54	Male			Diffused	Negative	gastric cancer
SRR7014404	61	Male			Diffused	Positive	gastric cancer
SRR7014405	51	Male			Diffused	Negative	gastric cancer
SRR7014406	48	Female			Diffused	Positive	gastric cancer
SRR7014407	52	Male			Diffused	Positive	gastric cancer
SRR7014408	73	Male			Diffused	Negative	gastric cancer

SRR7014409	53	Male			Diffused	Positive	gastric cancer
SRR7014410	51	Female			Diffused	Positive	gastric cancer
SRR7014411	69	Female			Diffused	Positive	gastric cancer
SRR7014412	69	Male			Diffused	Positive	gastric cancer
SRR7014413	81	Female			Diffused	Positive	gastric cancer
SRR7014414	82	Female			Diffused	Positive	gastric cancer
SRR7014415	51	Male			Diffused	Positive	gastric cancer
SRR7014416	79	Male			Diffused	Negative	gastric cancer
SRR7014417	36	Female			Diffused	Positive	gastric cancer
SRR7014418	55	Male			Diffused	Positive	gastric cancer
SRR7014419	62	Male			Diffused	Positive	gastric cancer
SRR7014420	85	Male			Diffused	Negative	gastric cancer
SRR7014421	61	Male			Diffused	Positive	gastric cancer
SRR7014422	36	Male			Diffused	Positive	gastric cancer
SRR7014423	76	Male			Diffused	Positive	gastric cancer
SRR7014424	48	Female			Diffused	Positive	gastric cancer
SRR7014425	71	Male			Diffused	Positive	gastric cancer
SRR7014426	83	Female			Diffused	Negative	gastric cancer
SRR7014427	67	Male			Diffused	Positive	gastric cancer
SRR7014428	72	Male			Diffused	Negative	gastric cancer
SRR7014429	46	Male			Diffused	Positive	gastric cancer
SRR7014430	66	Female			Diffused	Positive	gastric cancer

Supplementary Table 2 PCR Primer Sequences and Related Information

Number	Accession.versi	Virus	Species	Sense primer（5‘-3’）	Anti-sense primer（5‘-3’）
1	GU934326.1	RNA Virus	Acutely transforming retrovirus	5' AACTGGTGGTGGTTGGAGC 3'	5' AAGCCTTCGCCTGTCCTCA 3'
2	NC_022518.1	RNA Virus	Human endogenous retrovirus K	5' GCCATCCACCAAGAAGGCAGAG [illegible]	5' AGGGCGGGAAAGGCACATAG 3'
3	S52495.1	Phage	Phage 16	5' ATGGGTATTGCATAGGTG 3'	5' CTAATCAAGCATAGTGGC 3'
4	AB898927.1	RNA Virus	Feline leukemia virus	5' GTACTCCTTCCAGACCCACG 3'	5' CTTGATGCCCTCCTTGCAC 3'
5	MK948408.1	RNA Virus	Porcine type-C oncovirus	5' CTCGTTCCCGTTCTTATGCC 3'	5' TGCTTGCTGATGTGAAATGTC 3'
6	MN820536.1	RNA Virus	Phocid orthoreovirus 1	5' CAGGCTTACCGCATCTTCC 3'	5' GACCTTCCAAAGTAGGAGACG 3
7	KM209255.1	Phage	Dickeya phage phiDP10.3	5' GTAGTCCACGCTGTAAACGATG 3	5' CGAAGGCACTAAGGCATCT 3'
8	AF104025.1	RNA Virus	Pestivirus tauri	5' TCGTGGAACCCGTTTATG 3'	5' CACCGCTCCGTTCACATT 3'
9	MW355266.1	RNA Virus	Hepatitis E virus	5' CACTTGCTGTTGCTCCATATG 3'	5' GGCTGGTCGTCTGAATCTTG 3'

Supplementary Table 3 Marker gene of Single-cell transcriptome

Number	Cell type	Mark gene
1	T Cell	CD3D、CD3E、CD8A
2	B Cell	CD19、CD79A、MS4A1、CD20
3	Plasma Cell	IGHG1、MZB1、SDC1、CD79A
4	Monocyte and Macrophage	CD68、CD163、CD14
5	Fibroblast	FGF7、MME
6	Endothelial cell	PECAM1、VWF
7	Epithelial cell or Tumor	EPCAM、KRT19、PROM1、ALDH1A1、CD24
8	Neutrophil	FCGR3B
9	Smooth Muscle Cell	MYL9、RGS5
10	Mast Cell	TPSB2

Supplementary Table 4 HERV-K loci distribution

Number	Chromosome	Strand	Position start	Position end	Length (bp)	HERV-K Position start	HERV-K Position end	HERV-K Length (bp)	Gene	Intergenic/intron/exon
1	1	+	1409808	1410774	967	1	968	968	MRPL20 lincRNA	Intergenic
2	1	-	75377087	75380591	3505	1	3505	3505	SLC44A5	intron
3	1	-	150632876	150634622	1747	4788	6541	1754	ENSA	Intergenic
4	1	+	155626577	155635756	9180	1	9472	9180	LOC100419798	intron and exon
5	1	+	156179224	156180192	969	1	969	969	SEMA4A	exon
6	1	+	160690786	160700017	9232	1	9472	9180	CD48, LOC124904439	intron of CD48, exon of LOC124904439
7	1	+	207635111	207638347	3237	6048	9472	3425	CR1	intron
8	2	+	201139003	201139968	966	1	968	968	CFLAR	intron and exon
9	3	+	101691894	101701016	9123	1	9472	9180	NA	NA
10	3	-	113024260	113033418	9159	1	9472	9180	NEPRO-AS1	intron
11	3	+	125890460	125899574	9115	1	9293	9136	ENPP7P4	exon
12	3	-	148563597	148567524	3928	961	4887	3927	NA	NA
13	3	-	185561880	185571059	9180	1	9472	9180	NA	NA
14	4	+	160658787	160660938	2152	1	2320	2152	NA	NA
15	5	-	30486215	30495660	9446	1	9472	9472	NA	NA
16	5	-	156657697	156666878	9182	1	9472	9180	SGCD	intron
17	6	-	77716489	77725910	9422	1	9472	9472	MEI4	intron
18	7	-	4582425	4591896	9472	1	9472	9472	LOC124901580	Intergenic and exon
19	7	-	104748901	104752821	3921	969	4887	3919	LHFPL3	intron
20	8	-	7497874	7507336	9463	1	9472	9472	DEFB107B	intron
21	8	-	37193367	37194334	968	1	968	968	NA	NA
22	8	-	139459653	139460952	1300	947	2246	1300	NA	NA
23	10	-	6824178	6833640	9463	1	9472	9472	LINC00707, LOC105376387	intron and exon
24	10	-	99824891	99830987	6097	940	7038	6099	ABCC2	intron
25	11	+	101695064	101704529	9466	1	9472	9472	LOC124902738	exon
26	11	-	118720784	118729943	9160	1	9472	9180	LOC124902766	exon
27	12	-	58327458	58336914	9457	1	9472	9472	LOC105369787	intron and exon
28	16	-	34412056	34414726	2671	6794	9472	2679	NA	NA
29	16	+	34997104	34999772	2669	6794	9472	2679	NA	NA
30	19	-	27637589	27646474	8563	947	9472	8526	NA	NA
31	19	+	37106647	37116272	9472	1	9472	9472	ZNF420	intron
32	20	-	35262123	35263090	968	8505	9472	968	MMP24-AS1-EDEM2 and MMP24	intron
33	22	+	18938675	18947849	9175	1	9472	9180	LOC122455341(PRODH)	intron
34	Y	-	22893035	22894005	971	1	971	971	NA	NA
35	Y	+	25142349	25143316	968	1	968	968	NA	NA
